# Prognostic value of the non-high-density lipoprotein cholesterol to high-density lipoprotein cholesterol ratio (NHHR) in patients with RAS-mutant metastatic colorectal cancer

**DOI:** 10.3389/fnut.2025.1617930

**Published:** 2025-10-31

**Authors:** Wenxia Xie, Huizhuo Liu, Yunjiao Shi, Bingxin Zhang, Ruoyun Wang, Shayan Wang, Bin Liang

**Affiliations:** ^1^Department of Medical Oncology, First Affiliated Hospital of Wenzhou Medical University, Wenzhou, China; ^2^Department of Internal Medicine, First Affiliated Hospital of Wenzhou Medical University, Wenzhou, China

**Keywords:** metastatic colorectal cancer, Non-HDL-C/HDL-C, NHHR, prognosis, RAS-mutant

## Abstract

**Background:**

Patients with metastatic colorectal cancer (mCRC) generally have a poor prognosis, and treatment strategies are highly dependent on the RAS mutational status. During disease progression, patients often exhibit dynamic changes in serum lipid profiles. However, the prognostic significance of cholesterol-related biomarkers remains controversial. This study aimed to investigate the association between the non-high-density lipoprotein cholesterol to high-density lipoprotein cholesterol ratio (NHHR) and clinical outcomes in patients with RAS-mutant mCRC.

**Methods:**

This retrospective study included 287 RAS-mutant mCRC patients, all of whom received at least three cycles of chemotherapy combined with bevacizumab. Among them, 169 patients were assigned to the training cohort and 118 patients to the validation cohort. Pearson's correlation coefficient and Spearman's rank correlation test were used to assess the relationships between continuous variables. The optimal cutoff value for the NHHR was determined using the maximum selection rank statistic, categorizing patients into high and low NHHR groups. Kaplan-Meier survival curves were used to assess survival in the high and low NHHR groups, with differences between groups compared using the log-rank test. Restricted cubic splines (RCS) were employed to analyze the non-linear relationship between NHHR and mortality risk. Univariate and multivariate Cox regression models were used to assess the independence of NHHR in predicting survival outcomes, with stepwise adjustment for confounders and stratified analysis. A nomogram was constructed based on the final model.

**Results:**

After adjusting for confounders, the high NHHR group (>3.45) had a significantly higher mortality risk than the low NHHR group (HR = 2.23, 95% CI: 1.46–3.40, *P* < 0.001). Subgroup analyses revealed a stronger association in female patients (female, HR = 4.24, 95% CI: 1.85–9.71; male, HR = 1.72, 95% CI: 1.09–2.73; *P* for interaction = 0.037). The RCS analysis showed a linear increase in mortality risk with increasing NHHR (*P* for overall *P* < 0.001, *P* for non-linearity = 0.090). NHHR showed significant positive correlations with white blood cells, monocytes, neutrophils, fibrinogen, CA199, and CEA (all *P* < 0.05), and significant negative correlations with albumin, sodium, and chloride (all *P* < 0.05). The nomogram demonstrated robust predictive performance.

**Conclusion:**

NHHR may serve as a potential prognostic biomarker in patients with RAS-mutant metastatic colorectal cancer. Its putative role in promoting tumor progression through modulation of chronic inflammation warrants further investigation.

## Introduction

Colorectal cancer (CRC) is one of the most common malignant tumors worldwide. According to the Global Cancer Statistics 2022 (GLOBOCAN), CRC ranks third in terms of global cancer incidence and second in cancer-related mortality (1). In 2022, an estimated 1.926 million new CRC cases and ~904,000 CRC-related deaths were reported, accounting for 9.6% of all cancer diagnoses and 9.3% of cancer-related deaths, respectively (1). Despite significant advancements in colonoscopy screening and imaging techniques that have improved early detection rates, distant metastases are still present in 20%−25% of patients at the time of initial diagnosis (2, 3). Among patients initially diagnosed with a localized stage, follow-up studies have shown that ~18% develop local recurrence, 78% progress to distant metastasis, and 4% exhibit simultaneous recurrence and metastasis (3). The liver, lungs, and peritoneum are the most common sites of metastasis (4). In recent years, the clinical application of targeted therapies, immunotherapy, and adoptive cell therapy (ACT) has offered new hope for improving the prognosis of patients with advanced metastatic colorectal cancer (mCRC) (5, 6). However, the 5-year survival rate for patients with mCRC remains ~14%, which is markedly lower than the 89% observed in patients with localized disease (7).

Colorectal cancer treatment strategies are closely related to RAS gene status. RAS wild-type patients respond well to cetuximab, while RAS mutant patients exhibit primary resistance (8). For RAS mutant patients, clinical guidelines recommend fluorouracil-based chemotherapy combined with bevacizumab (9). However, clinical observations reveal significant prognostic heterogeneity even with standard treatment, suggesting that other potential regulatory mechanisms, beyond genetic mutations, remain unexplored. In recent years, with the advancement of metabolomics, biomarkers related to metabolic reprogramming and serum lipid profiles have gained attention in cancer prognosis (10, 11). Systematically investigating the correlation between these novel biological markers and CRC prognosis is crucial for optimizing clinical treatment strategies and advancing personalized medicine.

Cholesterol and its associated lipoproteins serve as crucial bioactive molecules involved in key pathophysiological processes, including the regulation of cell membrane homeostasis, signal transduction, and inflammation modulation (12). High-density lipoprotein cholesterol (HDL-C) is recognized as a cardiovascular protective factor due to its role in mediating reverse cholesterol transport (13). Recent studies have further demonstrated that HDL-C may exert tumor-suppressive effects by modulating oxidative stress, immune microenvironment, and anti-inflammatory pathways (14). In contrast, non-high-density lipoprotein cholesterol (non-HDL-C), which comprises low-density lipoprotein cholesterol (LDL-C) and very-low-density lipoprotein cholesterol (VLDL-C), consists of atherogenic lipoprotein particles. Its pro-tumorigenic effects may be attributed to mechanisms such as the activation of proliferative signaling pathways, enhancement of metastatic potential, and inhibition of apoptosis (15, 16). Given these opposing biological properties, the non-HDL-C/HDL-C ratio (NHHR) has emerged as a novel prognostic biomarker reflecting lipid metabolism homeostasis, garnering increasing attention across various research fields in recent years.

NHHR has been associated with the incidence of various diseases, including diabetes (17), kidney stones (18), osteoporosis (19), and hyperuricemia (20). Additionally, NHHR has been proposed as an indicator for monitoring and preventing sarcopenia in cancer patients (21). NHHR is not only associated with disease risk but also with disease mortality. Studies have shown that elevated NHHR correlates with a heightened risk of mortality in patients with sepsis (22). Among U. S. adults with diabetes or prediabetes, a U-shaped relationship was observed between NHHR and all-cause mortality, while an L-shaped association was noted with cardiovascular mortality (23).

Epidemiological studies have shown that abnormalities in lipid metabolism are associated with a range of malignancies, including breast (24), endometrial (25), lung (26), liver (27), esophageal (28), gastric (29), colorectal (30), and hematologic cancers (31). In colorectal cancer, most studies indicate that lower HDL-C levels significantly elevate cancer risk, whereas reduced levels of non-HDL-C, particularly LDL-C, are associated with a decreased risk of cancer (32–36). Although pan-cancer analyses have suggested a potential J-shaped association between NHHR and all-cause mortality (37), its prognostic significance in patients with RAS-mutant mCRC remains unclear.

This study aims to systematically evaluate the relationship between NHHR and prognosis in patients with RAS-mutant mCRC. Through clinical data analysis and multivariable adjustments, we seek to elucidate the independent role of the NHHR in mCRC prognosis and explore its potential biological mechanisms. Our findings will contribute to a more profound comprehension of the role of cholesterol dysregulation in CRC progression and offer new insights and strategies for clinical treatment and management.

## Methods

### Patient selection

**Figure 1:**
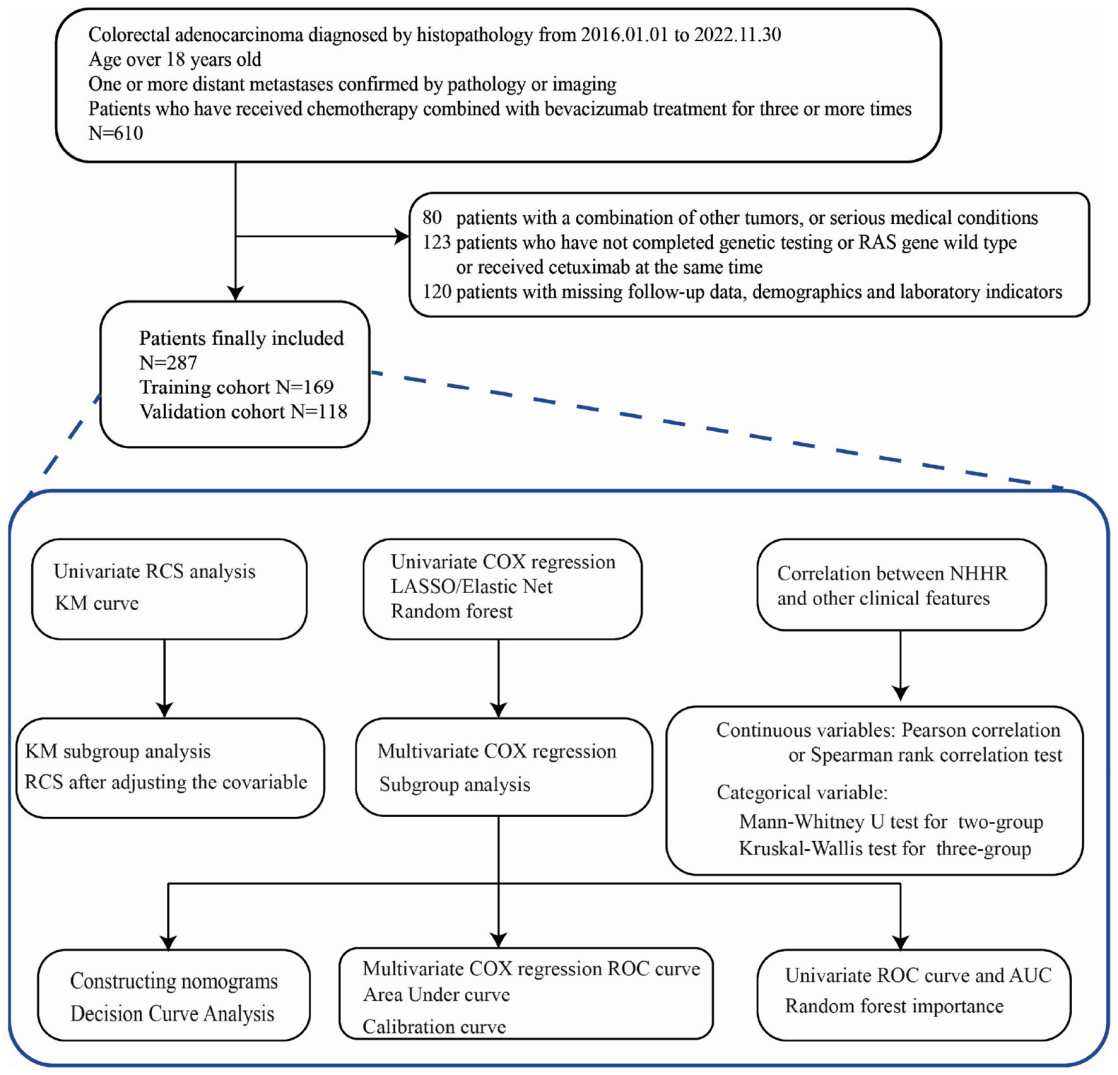
Flowchart of the selection process and study design.

This retrospective study analyzed patients with RAS-mutant mCRC who received at least three cycles of chemotherapy combined with bevacizumab at the First Affiliated Hospital of Wenzhou Medical University between January 1, 2016, and November 30, 2022. The detailed patient selection process is illustrated in Figure 1.

The inclusion criteria were as follows: (1) age ≥ 18 years; (2) histologically confirmed colorectal adenocarcinoma; (3) evidence of one or more distant metastases confirmed by pathology or imaging; and (4) treatment with at least three cycles of standard chemotherapy combined with bevacizumab at our institution.

Exclusion criteria included: (1) missing follow-up data, demographic information, or laboratory results; (2) concomitant malignancies or severe comorbidities (e.g., heart, liver, lung, or renal failure, severe infections, or coagulation disorders); and (3) incomplete RAS mutation testing, RAS wild-type status, or concurrent treatment with cetuximab.

A total of 610 patients initially met the diagnostic criteria. Of these, 80 were excluded due to coexisting malignancies or serious comorbidities. Another 123 patients were excluded for incomplete RAS testing, RAS wild-type status, or concurrent cetuximab treatment. Additionally, 120 patients were excluded due to missing follow-up, demographic, or laboratory data. Ultimately, 287 patients were included in the final analysis. Among them, 169 cases are divided into the training set, and 118 cases are divided into the validation set.

### Data collection

We obtained relevant information from the hospital information system (HIS) of the First Affiliated Hospital of Wenzhou Medical University (https://dc.wzhospital.cn/vpn/index.html).

General information: patient's name, case number, date of birth, time of admission, sex, age; any comorbidity with hypertension, diabetes mellitus or other major diseases. History of smoking and drinking, height and weight at the time of admission. Use of lipid-lowering medications (e.g., statins, ezetimibe, fibrates) during the treatment period was also recorded.

Tumor characteristics: initial symptoms of the tumor, RAS mutation status, stage, pathological grading, whether distant metastasis and metastatic sites, whether previous colorectal cancer surgery, time of surgery, and type of surgery (radical or non-radical surgery), number of previous lines of chemotherapy, and whether radiotherapy was received.

Laboratory parameters: all blood samples were collected as fasting blood within 1 week before enrollment under standardized conditions. The tested parameters included blood routine indicators such as peripheral blood lymphocyte count (Lym), neutrophil count (Neu), platelet count (Plt), monocyte count (Mono), and red blood cell distribution width (RDW); liver function indicators including globulin (GLB), albumin (ALB), alkaline phosphatase (ALP), and total bilirubin (TBIL); renal function indicators such as creatinine (Cr), uric acid (UA), and urea nitrogen (BUN); tumor markers including carcinoembryonic antigen (CEA), carbohydrate antigen 199 (CA199), and alpha-fetoprotein (AFP); coagulation indicators such as fibrinogen (FIB) and D-dimer (D-D); biochemical parameters including creatine kinase (CK), potassium (K+), chloride (Cl-), sodium (Na+), and glucose (Glu); and lipid indicators such as high-density lipoprotein cholesterol (HDL-C), low-density lipoprotein cholesterol (LDL-C), total cholesterol (TC), and triglycerides (TG).

### Relevant definitions

NHHR is quantified as the ratio of non-HDL-C to HDL-C. The calculation formula is as follows: NHHR = (TC-HDL-C)/HDL-C. Body mass index (BMI) is calculated as body weight (kg) divided by the square of height (m^2^). Tumor histological grades are classified into four levels: grade 1 (well-differentiated), grade 2 (moderately differentiated), grade 3 (poorly differentiated), and grade 4 (undifferentiated). Tumor metastasis is categorized into three stages: a, b, and c. A indicates a single site of distant metastasis, where the tumor has spread to a single distant organ or tissue (e.g., liver, lung), but the metastasis is limited to one site. B refers to the spread to multiple distant organs or tissues without peritoneal involvement. C indicates metastasis not only to one or more distant organs but also to the peritoneum. Blood in the stool, change of character, frequency, urgency, and difficulty in defecation were defined as change of stool character; abdominal pain, abdominal discomfort, mass, weight loss, intestinal obstruction, and other lymphatic metastases were defined as other signs; asymptomatic was defined as the absence of any symptoms and signs, which indicated that they were detected only by physical examination (including elevated tumor markers, imaging abnormalities, and colonoscopy abnormalities, etc.). The tumor sites were divided into the left half of the colon (including the descending colon, sigmoid colon, and rectum) and the right half of the colon (including the ascending colon and transverse colon) according to the splenic flexure. The median continuous variable was used as a cutoff value to divide into higher and lower groups when performing subgroup analyses.

In this study, RAS mutation status was primarily determined by reverse transcriptase-polymerase chain reaction (RT-PCR) and Sanger sequencing during the sample collection period, when next-generation sequencing (NGS) was still in the early stages of clinical adoption. Overall, KRAS mutations accounted for 89.2% of cases, whereas NRAS mutations comprised 10.8%. Detailed codon-specific information was available only for a small subset of patients, in whom the most common variants were KRAS p.G12D, p.G13D, and p.G12V (Supplementary Figure S1).

### Follow-up

The primary endpoint of this study was overall survival (OS). The start date for OS was defined as the date of the patient's first hospitalization for mCRC at our institution, with patient death serving as the endpoint. Follow-up data were collected through a combination of electronic medical record reviews and telephone interviews. The last follow-up date was June 30, 2025.

### Ethics

The study was approved by the Ethics Committee of the First Affiliated Hospital of Wenzhou Medical University (KY2024-R293) and conducted in accordance with local legislation and institutional requirements. As a retrospective study using fully anonymized data, with no identifiable information or risks to participants, the Ethics Committee approved a waiver of informed consent.

This waiver is consistent with Article 32 of the Declaration of Helsinki (2013), which permits the use of identifiable human data without informed consent when obtaining it is impracticable and the research carries minimal risk, provided that ethics approval has been obtained. It also aligns with Article 12 of the “Administrative Measures for Ethical Review of Biomedical Research Involving Human Subjects (Trial)” issued by the National Health and Family Planning Commission of China, which allows ethics committees to exempt informed consent requirements for studies using existing clinical data that do not involve personal privacy.

### Statistical analysis

Data analysis was performed using SPSS version 26.0, while R software (version 4.4.1) was employed for supplementary analyses and graphing. Adobe Illustrator 2023 was used for image composition. The normality of continuous variables was evaluated using the Kolmogorov-Smirnov test (Supplementary Table S1), along with histograms and Q–Q plots. Continuous variables with a normal distribution were expressed as mean ± standard deviation (SD), and comparisons between two groups were made using independent samples *t*-tests. For non-normally distributed variables, data were presented as median (Q1, Q3), and comparisons between groups were conducted using the Mann–Whitney *U* test or Kruskal–Wallis test for two or three independent samples, respectively. Categorical variables were reported as frequencies (percentages) and compared using the chi-square test.

The optimal NHHR cutoff associated with survival outcomes was identified using the maximum selection rank statistic from the ‘maxstat' package, dividing participants into higher- and lower-NHHR groups. Kaplan-Meier survival curves and log-rank tests were applied to assess survival probabilities at different NHHR levels. Three multivariable Cox regression models were developed to adjust for potential confounders. Model 1 adjusted for NHHR, age, sex, BMI, hypertension, diabetes, and smoking/drinking history. Model 2 further included tumor histological grade, tumor location, metastasis, initial symptoms, and treatment history (surgery, chemotherapy, radiotherapy). Model 3 additionally accounted for laboratory markers, including CEA, CA199, albumin, and lymphocyte levels.

Univariate Cox regression analyses were first performed on all clinical and laboratory variables, and those associated with survival at *P* < 0.1 were considered candidates for further evaluation. Given the high dimensionality of laboratory parameters, complementary approaches were applied to enhance variable selection. Penalized regression techniques, including the least absolute shrinkage and selection operator (LASSO) and Elastic Net, were implemented with 10-fold cross-validation to shrink coefficients and identify prognostically relevant variables. In parallel, survival random forest was used to assess variable importance based on the mean decrease in Gini index. To ensure clinical interpretability and robustness, variables consistently retained across statistical methods, along with clinically meaningful factors supported by prior evidence, were incorporated into the final multivariable Cox proportional hazards model. Significant predictors (*P* < 0.05) were subsequently used to construct a prognostic nomogram estimating 1-, 2-, and 3-year survival probabilities. Additionally, time-dependent receiver operating characteristic (ROC) curve analysis and calibration curves were performed to assess the model's accuracy in predicting survival outcomes. The clinical decision-making value of the model was evaluated using decision curve analysis (DCA).

The potential non-linear relationships between NHHR and various laboratory indicators associated with mortality risk were visualized using restricted cubic splines (RCS). The optimal number of knots was determined based on the minimum Akaike Information Criterion (AIC) or Bayesian Information Criterion (BIC) values, while the Wald chi-square test was utilized to assess the significance of non-linearity. Scatter plots were generated to illustrate the linear relationships between pairs of continuous variables. To investigate the linear correlations between NHHR and other blood parameters, Pearson correlation tests were employed for normally distributed data, and Spearman rank correlation tests were applied for non-normally distributed data.

In assessing risk factors, univariate Cox regression models were employed to analyze NHHR, CEA, age, BMI, lymphocyte, metastasis, chemotherapy, and surgery, while time-dependent ROC curves were used to calculate the Area Under the Curve (AUC) values for evaluating the contribution of each factor to predicting survival outcomes. Random forest models were subsequently applied to calculate Gini importance to further assess the relative importance of these factors. To assess the generalizability and robustness of the NHHR-based prognostic model, we applied the model to an independent validation cohort, and evaluated its performance using ROC analysis, calibration plots, DCA, and Kaplan–Meier survival curves. Unless otherwise specified, group comparisons were performed using two-tailed tests, with *P* < 0.05 considered statistically significant.

## Results

### Demographic and clinical characteristics distribution of RAS-mutant mCRC patients grouped by NHHR level

**Figure 2:**
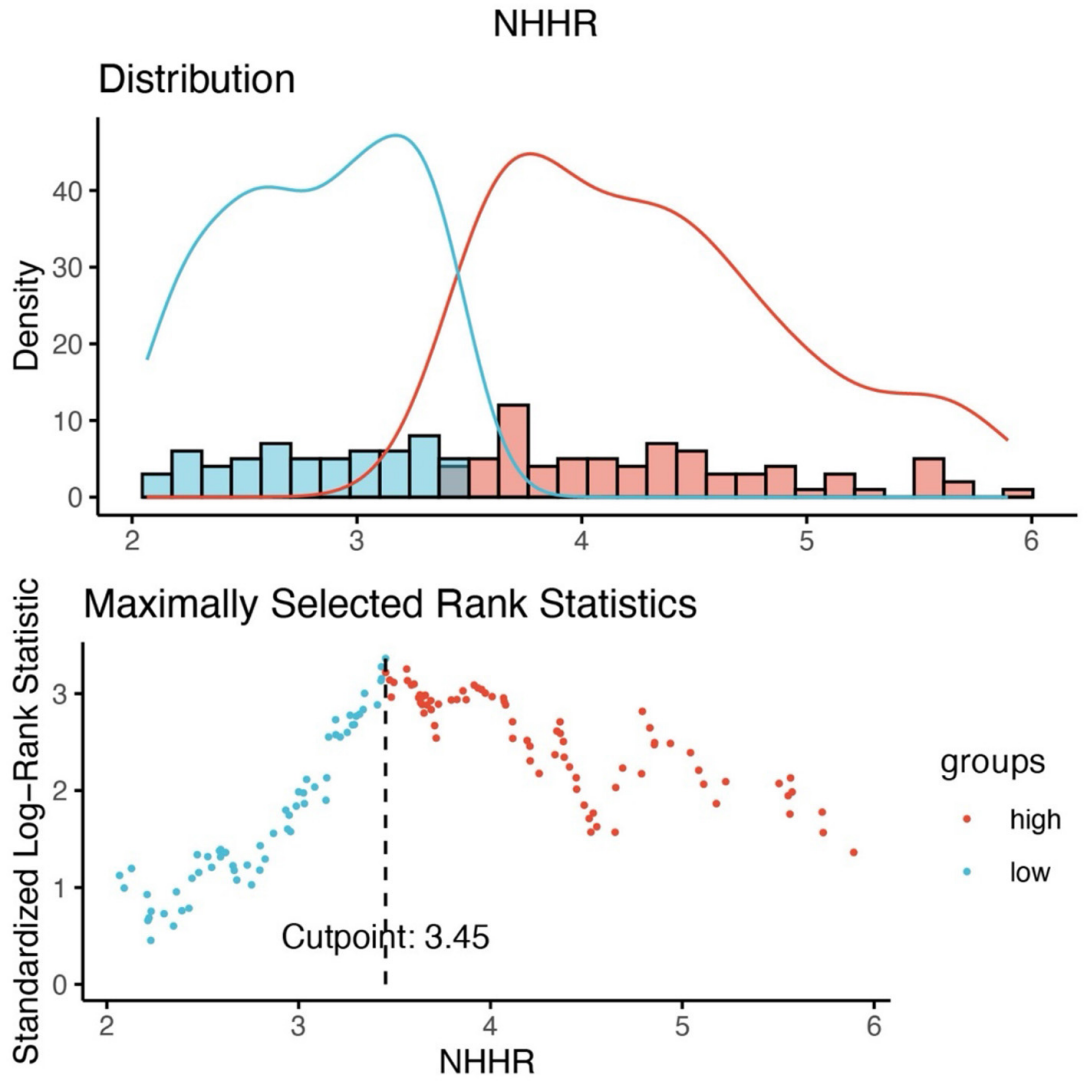
Calculate the optimal cutoff point based on the maximum selective rank statistic.

Based on the optimal NHHR cutoff of 3.45, patients were categorized into a high NHHR group (NHHR > 3.45, *n* = 92) and a low NHHR group (NHHR ≤ 3.45, *n* = 77) (Figure 2). Demographic and clinical characteristics of the two groups were compared (Table 1). Patients in the high NHHR group had significantly higher levels of TC, TG, LDL-C, and non-HDL-C, and significantly lower levels of HDL-C (all *P* < 0.001). CA199 levels were also significantly elevated in the high NHHR group (*P* = 0.028). In contrast, changes in stool characteristics as an initial symptom were more frequently reported in the low NHHR group (*P* = 0.010). Additionally, a higher proportion of patients in the low NHHR group reported a history of alcohol consumption (*P* = 0.023). No statistically significant differences were observed between the two groups in terms of age, sex, BMI, lymphocyte count, albumin, CEA levels, RAS mutation status, lipid-lowering medication use, metastatic sites, or treatment strategies (all *P* > 0.05).

**Table 1:** Characteristics of high and low NHHR study populations.

**Variables**	**Total (*n* = 169)**	**Lower NHHR (*n* = 77)**	**Higher NHHR (*n* = 92)**	**Statistic**	***P***
Age (years), Mean ± SD	60.76 ± 9.80	59.94 ± 10.78	61.45 ± 8.91	*t* = −1.00	0.320
BMI (kg/m^2^), Mean ± SD	22.83 ± 3.21	22.50 ± 3.38	23.12 ± 3.05	*t* = −1.24	0.215
TC (mmol/L), M (Q1, Q3)	4.95 (4.21, 5.61)	4.37 (3.81, 5.13)	5.31 (4.66, 5.97)	*Z* = −4.94	< 0.001
TG (mmol/L), M (Q1, Q3)	1.42 (1.07, 2.02)	1.27 (0.84, 1.69)	1.63 (1.24, 2.26)	*Z* = −3.69	< .001
LDL-C (mmol/L), M (Q1, Q3)	2.98 (2.32, 3.47)	2.49 (2.15, 2.97)	3.37 (2.73, 3.84)	*Z* = −6.23	< 0.001
HDL-C (mmol/L), M (Q1, Q3)	1.05 (0.90, 1.32)	1.29 (1.02, 1.48)	0.96 (0.79, 1.10)	*Z* = −6.51	< 0.001
Non-HDL-C (mmol/L), M (Q1, Q3)	3.79 (3.13, 4.45)	3.29 (2.64, 3.73)	4.22 (3.79, 5.01)	*Z* = −7.38	< 0.001
NHHR, M (Q1, Q3)	3.60 (2.80, 4.51)	2.66 (2.21, 3.08)	4.43 (3.85, 5.55)	*Z* = −11.18	< 0.001
Lym ( × 10^9^/L), M (Q1, Q3)	1.59 (1.18, 1.92)	1.52 (1.18, 1.84)	1.62 (1.17, 2.04)	*Z* = −1.11	0.266
ALB (g/L), M (Q1, Q3)	41.00 (37.60, 44.00)	41.90 (37.50, 45.40)	40.35 (37.77, 43.40)	*Z* = −1.57	0.116
CEA (ng/mL), M (Q1, Q3)	18.00 (4.20, 94.30)	14.60 (2.80, 78.30)	21.35 (7.65, 107.82)	*Z* = −1.92	0.055
CA199 (ng/mL), M (Q1, Q3)	17.20 (5.80, 111.00)	14.40 (3.50, 52.50)	24.85 (8.57, 177.42)	*Z* = −2.19	0.028
**Metastasis**, ***n*** **(%)**					
a	71 (42.01)	32 (41.56)	39 (42.39)	χ^2^ = 0.06	0.970
b	36 (21.30)	16 (20.78)	20 (21.74)		
c	62 (36.69)	29 (37.66)	33 (35.87)		
**Sex**, ***n*** **(%)**					
Female	54 (31.95)	30 (38.96)	24 (26.09)	χ^2^ = 3.20	0.074
Male	115 (68.05)	47 (61.04)	68 (73.91)		
**Grade**, ***n*** **(%)**					
1	25 (14.79)	14 (18.18)	11 (11.96)	χ^2^ = 3.10	0.212
2	80 (47.34)	31 (40.26)	49 (53.26)		
3/4	64 (37.87)	32 (41.56)	32 (34.78)		
**Location**, ***n*** **(%)**					
Left half of the colon (rectum)	127 (75.15)	56 (72.73)	71 (77.17)	χ^2^ = 0.44	0.505
Right half of the colon	42 (24.85)	21 (27.27)	21 (22.83)		
**Initial symptoms**, ***n*** **(%)**					
Changes in stool properties	108 (63.91)	53 (68.83)	55 (59.78)	χ^2^ = 9.15	0.010
Other signs	49 (28.99)	15 (19.48)	34 (36.96)		
Asymptomatic	12 (7.10)	9 (11.69)	3 (3.26)		
**Chemotherapy**, ***n*** **(%)**					
First-line	35 (20.71)	14 (18.18)	21 (22.83)	χ^2^ = 1.18	0.554
Second-line	58 (34.32)	25 (32.47)	33 (35.87)		
More than three lines	76 (44.97)	38 (49.35)	38 (41.30)		
**Hypertension**, ***n*** **(%)**					
NO	98 (57.99)	49 (63.64)	49 (53.26)	χ^2^ = 1.85	0.174
YES	71 (42.01)	28 (36.36)	43 (46.74)		
**Diabetes**, ***n*** **(%)**					
NO	138 (81.66)	64 (83.12)	74 (80.43)	χ^2^=0.20	0.654
YES	31 (18.34)	13 (16.88)	18 (19.57)		
**Smoke**, ***n*** **(%)**					
NO	117 (69.23)	50 (64.94)	67 (72.83)	χ^2^ = 1.23	0.268
YES	52 (30.77)	27 (35.06)	25 (27.17)		
**Drinking**, ***n*** **(%)**					
NO	124 (73.37)	50 (64.94)	74 (80.43)	*χ*^2^ = 5.15	0.023
YES	45 (26.63)	27 (35.06)	18 (19.57)		
**Radiation therapy**, ***n*** **(%)**					
NO	143 (84.62)	65 (84.42)	78 (84.78)	χ^2^ = 0.00	0.947
YES	26 (15.38)	12 (15.58)	14 (15.22)		
**Surgery**, ***n*** **(%)**					
NO	24 (14.20)	7 (9.09)	17 (18.48)	χ^2^ = 3.03	0.082
YES	145 (85.80)	70 (90.91)	75 (81.52)		
**RAS mutation subtypes**, ***n*** **(%)**					
KRAS	151 (89.35)	70 (90.91)	81 (88.04)	χ^2^ = 0.36	0.548
NRAS	18 (10.65)	7 (9.09)	11 (11.96)		
**Lipid lowering drugs**, ***n*** **(%)**					
NO	136 (80.47)	61 (79.22)	75 (81.52)	χ^2^ = 0.14	0.707
YES	33 (19.53)	16 (20.78)	17 (18.48)		

*t*: *t*-test, *Z*: Mann–Whitney test, χ^2^: Chi-square test, SD: standard deviation, M: median, Q1: 1st Quartile, Q3: 3st Quartile, BMI, body mass index; TC, total cholesterol; TG, triglycerides; LDL-C, low-density lipoprotein cholesterol; HDL-C, high-density lipoprotein cholesterol; NHHR, ratio of non-high-density lipoprotein cholesterol to high-density lipoprotein cholesterol; Lym, peripheral blood lymphocyte count; ALB, albumin; CEA, carcinoembryonic antigen; CA199, carbohydrate antigen 199; a: metastasis to a single distant organ or site (e.g., liver, lung), limited to one location; b: metastasis to multiple distant organs or sites, without peritoneal involvement; c: metastasis to one or more distant organs with peritoneal involvement.

### Correlation analysis between NHHR and laboratory indicators

**Figure 3:**
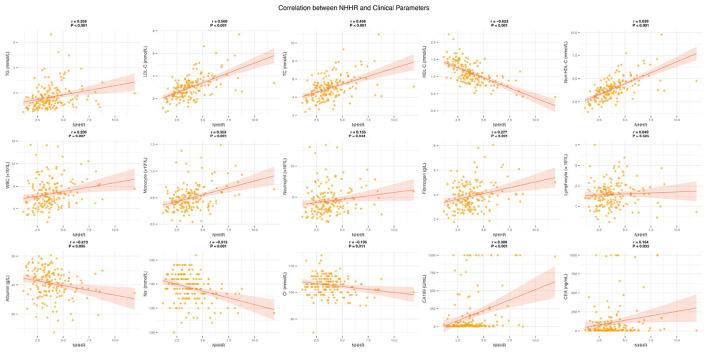
Correlation between NHHR and clinical parameters.

We performed an in-depth analysis of the potential correlations between NHHR and various clinical variables using correlation scatterplot matrices, boxplots, and kernel density estimate (KDE) plots (Supplementary Figure S2). The results showed that NHHR was significantly positively correlated with TG, LDL-C, and TC, while it was significantly negatively correlated with HDL-C (all *P* < 0.05). Among peripheral blood parameters, NHHR exhibited significant positive correlations with WBC, monocytes, neutrophils, and fibrinogen (all *P* < 0.05). Although NHHR showed a weak positive correlation with lymphocytes, this was not statistically significant (*P* = 0.525). Additionally, NHHR was significantly negatively correlated with albumin, Na^+^, and Cl^−^ levels (all *P* < 0.05). Notably, the cancer markers CA199 and CEA were positively correlated with NHHR, with correlation coefficients of 0.308 (*P* < 0.001) and 0.164 (*P* < 0.05), respectively (Figure 3). We also compared NHHR values across different clinical subgroups (Supplementary Figure S3). The results showed that NHHR levels in the surgery group were significantly lower than in the non-surgery group (*P* = 0.006). In contrast, no significant differences in NHHR were observed across other clinical characteristic subgroups.

### Exploring the non-linear relationship between NHHR and mortality using restricted cubic splines

**Figure 4:**
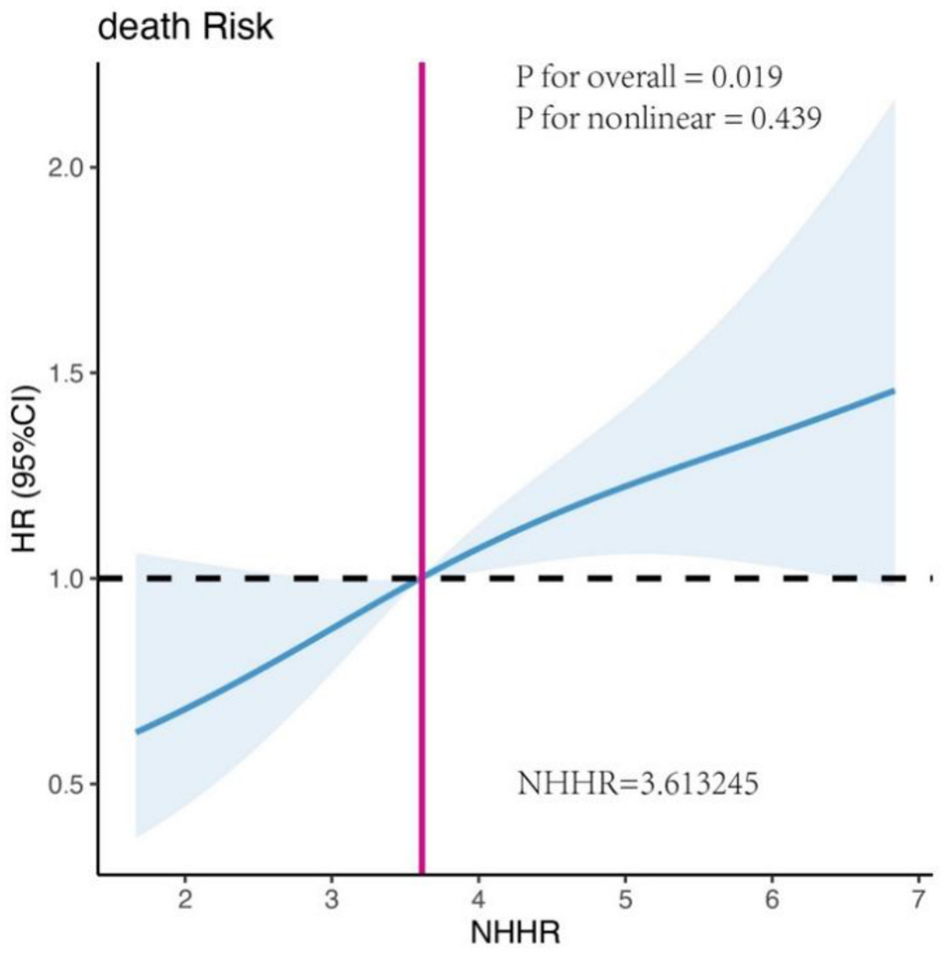
Univariate restricted cubic spline (RCS) analysis of the association between NHHR and mortality risk in patients with RAS-mutated mCRC.

Univariate RCS analysis revealed a positive linear association between NHHR and mortality in RAS-mutant mCRC patients (*P* for overall = 0.019, *P* for non-linearity = 0.439) (Figure 4). The hazard ratio (HR) curve for HDL-C showed a non-linear trend with survival, although it remained relatively stable (*P* for overall = 0.047, *P* for non-linearity = 0.064) (Supplementary Figure S4A). In contrast, the HR curve for non-HDL-C exhibited a slight upward trend, suggesting that increasing non-HDL-C levels were associated with a higher mortality risk, although this change was not statistically significant (*P* for overall = 0.075, *P* for non-linearity = 0.080) (Supplementary Figure S4B). Other lipid metabolism indicators, including LDL-C, TC, and TG, were not significantly associated with mortality risk (all *P* for overall > 0.05) (Supplementary Figures S4C–E). However, elevated tumor markers (CEA, CA199, AFP) were significantly associated with higher mortality risk (CEA: *P* for overall = 0.001, *P* for non-linearity = 0.014; CA199: *P* for overall = 0.018, *P* for non-linearity = 0.034; AFP: *P* for overall = 0.011, *P* for non-linearity = 0.008) (Supplementary Figures S4H–J). Additionally, higher levels of lymphocytes and albumin were significantly associated with lower mortality risk (Lym: *P* for overall = 0.003, *P* for non-linearity = 0.005; ALB: *P* for overall = 0.033, *P* for non-linearity = 0.115) (Supplementary Figures S4F, G).

### Association between NHHR and survival: Kaplan–Meier curve

**Figure 5:**
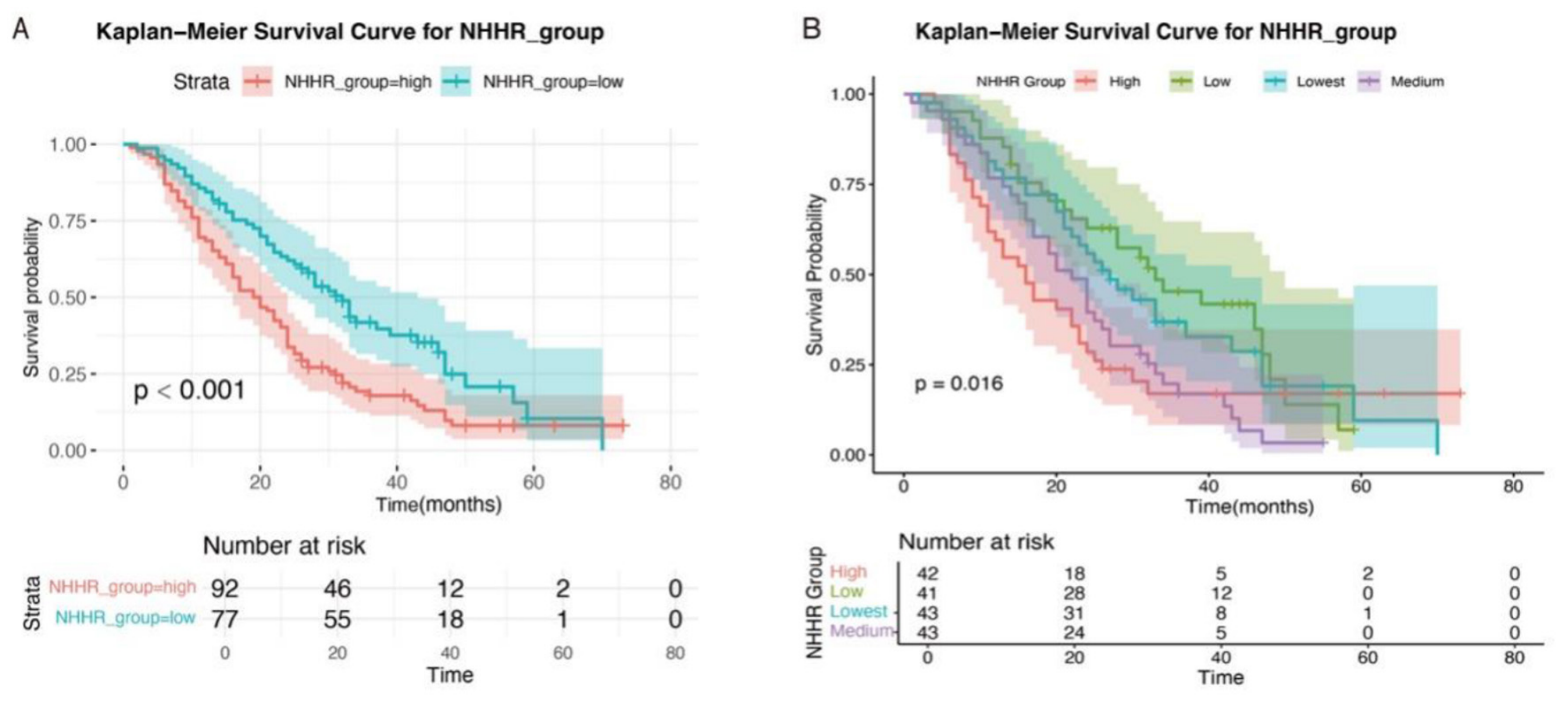
Kaplan–Meier survival curves of RAS-mutant mCRC patients stratified by NHHR levels. **(A)** Classification of NHHR into high and low groups based on cutoff values. **(B)** Classification of NHHR into high, medium, low, and lowest groups based on quartiles.

During a median follow-up period of 24 months, a total of 132 deaths were recorded among 169 patients with RAS-mutant mCRC. Survival analysis revealed that the 1-, 2-, and 3-year overall survival rates were ~75.7%, 45.5%, and 28.6%, respectively. Kaplan–Meier survival analysis demonstrated that patients in the high NHHR group had significantly higher mortality than those in the low NHHR group (log-rank test, *P* < 0.001; Figure 5A).

To further evaluate the association between NHHR and prognosis, patients were additionally stratified into four subgroups according to NHHR quartiles: high, medium, low, and lowest (Figure 5B). Significant differences in survival probabilities were still observed among the groups (*P* = 0.016). Moreover, subgroup analyses using Kaplan–Meier survival curves based on various clinical characteristics consistently showed worse survival outcomes in the high NHHR group across all subgroups (Supplementary Figure S5). There was no statistically significant difference in overall survival between patients with and without lipid-lowering drug use (Log-rank *P* = 0.202), or between those with KRAS and NRAS mutations (Log-rank *P* = 0.430) (Supplementary Figure S6A).

### NHHR is an independent prognostic factor for RAS-mutant mCRC patients

To evaluate the independent prognostic value of NHHR as a predictive factor for mortality in patients with RAS-mutant mCRC, we constructed three Cox proportional hazards models with progressive adjustment for potential confounders, including demographic characteristics, tumor-related features, and laboratory indicators (Table 2). In the unadjusted model, an increase in NHHR was significantly associated with a higher risk of death (HR = 1.15, 95% CI: 1.04–1.27). After multivariable adjustment, each one-unit increase in NHHR was associated with a 17%, 17%, and 19% increased risk of mortality in Models 1, 2, and 3, respectively. Furthermore, we performed multivariable Cox regression analyses by treating NHHR as a categorical variable. In the unadjusted model, patients in the high NHHR group had a significantly increased risk of death compared to those in the low NHHR group (HR = 1.80, 95% CI: 1.27–2.56). After adjusting for confounding variables, the risk of death in the high NHHR group increased by 83%, 111%, and 123% in Models 1, 2, and 3, respectively (Table 2). In addition, we explored the association between the use of lipid-lowering drugs and prognosis. Although patients receiving lipid-lowering treatment showed a trend of improved survival rates (Supplementary Table S2), this association did not reach statistical significance after adjusting for other covariates (Model 1: HR = 0.61 (95% CI: 0.37–1.01), Model 2: HR = 0.65 (95% CI: 0.38–1.10), Model 3: HR = 0.65 (95% CI: 0.38–1.10), Supplementary Tables S3–S5).

**Table 2:** Association between NHHR and mortality risk in RAS-mutant mCRC patients: multivariable cox regression analysis.

**Characteristic**	**Crude model**	***p***	**Model 1**	***p***	**Model 2**	***p***	**Model 3**	***p***
**Mortality**	**HR (95% CI)**		**HR (95% CI)**		**HR (95% CI)**		**HR (95% CI)**	
NHHR (continuous)	1.15 (1.04, 1.27)	0.005	1.17 (1.06, 1.30)	0.002	1.17 (1.05, 1.31)	0.005	1.19 (1.06, 1.34)	0.003
**NHHR category**								
Lower NHHR (*n* = 92)	Ref		Ref		Ref		Ref	
Higher NHHR (*n* =77)	1.80 (1.27, 2.56)	0.001	1.83 (1.27, 2.65)	0.001	2.11 (1.40, 3.19)	< 0.001	2.23 (1.46, 3.40)	< 0.001

The crude model was unadjusted. Model 1 was adjusted for demographic characteristics and comorbidities, including NHHR, age, sex, BMI, hypertension, diabetes, Lipid-lowering drugs and smoking/alcohol consumption history. Model 2 was further adjusted for tumor-related characteristics based on Model 1, including histological grade, primary tumor site, sites of metastasis, initial symptoms, RAS mutation subtype, and treatment history (surgery, chemotherapy, and radiotherapy). Model 3 included additional laboratory markers based on Model 2, including CEA, CA199, albumin, and lymphocyte levels.

### Screening and integration of prognostic variables for multivariate COX regression modeling.

**Figure 6:**
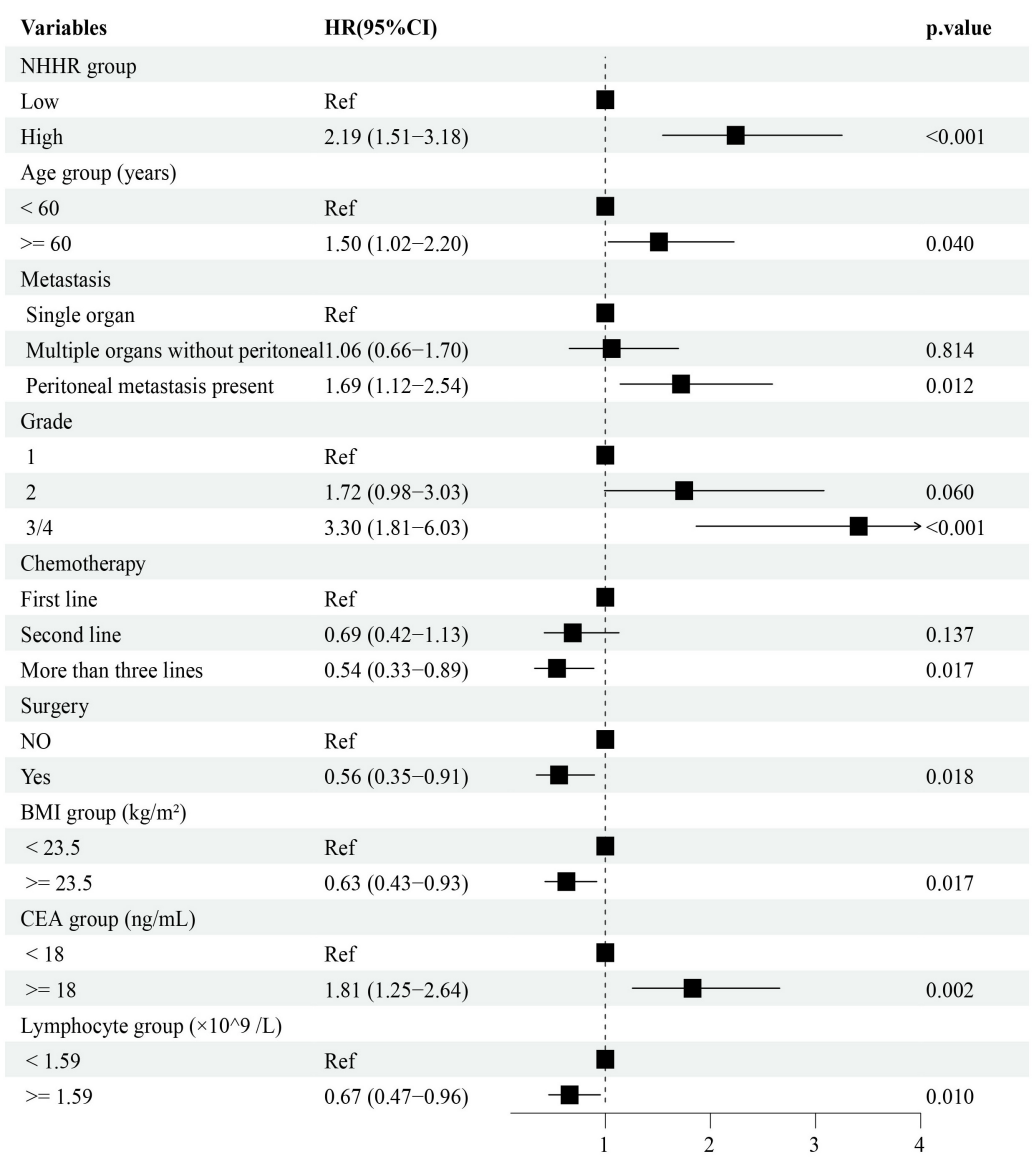
Multivariable Cox proportional hazards model of prognostic factors. NHHR, ratio of non-high-density lipoprotein cholesterol to high-density lipoprotein cholesterol.

Univariate Cox regression was first performed to screen for potential prognostic variables (*P* < 0.1; Supplementary Table S2). To further refine feature selection, LASSO, Elastic Net, and survival random forest analyses were performed (Supplementary Figure S7), which consistently highlighted NHHR as a robust prognostic factor, along with age, alkaline phosphatase, Cl^−^, and lymphocyte count. These variables, together with clinically meaningful covariates, were subsequently included in the multivariate Cox regression model, and those with *P* < 0.05 were ultimately incorporated into the prognostic prediction model. The results indicated that NHHR, CEA, age, BMI, lymphocyte count, site of metastasis, tumor histological grade, number of chemotherapy lines, and surgical intervention were all independently associated with overall survival. Specifically, elevated NHHR levels, presence of peritoneal metastasis, higher tumor histological grade, older age, and increased CEA levels were associated with poor prognosis, whereas higher BMI, increased lymphocyte count, multi-line chemotherapy, and surgery were identified as protective factors (Figure 6).

All included variables had variance inflation factors (VIFs) below 5, suggesting that multicollinearity was not a significant concern and could be considered negligible (Supplementary Table S6). In the multivariate Cox regression analysis including all patients (Figure 6), NHHR was found to be significantly and independently associated with poorer survival outcomes (HR = 2.19, 95% CI: 1.51–3.18, *P* < 0.001). Subgroup analyses based on clinical characteristics demonstrated that elevated NHHR remained positively associated with mortality risk across most subgroups (Supplementary Figure S8). Notably, the hazard ratio for female patients was significantly higher than for male patients (female: HR = 4.24, 95% CI: 1.85–9.71; male: HR = 1.72, 95% CI: 1.09–2.73), and a statistically significant interaction effect was observed between NHHR and sex on survival outcomes (interaction *P* = 0.037).

### Importance of NHHR as a biomarker for predicting survival in patients with RAS-mutant mCRC

**Figure 7:**
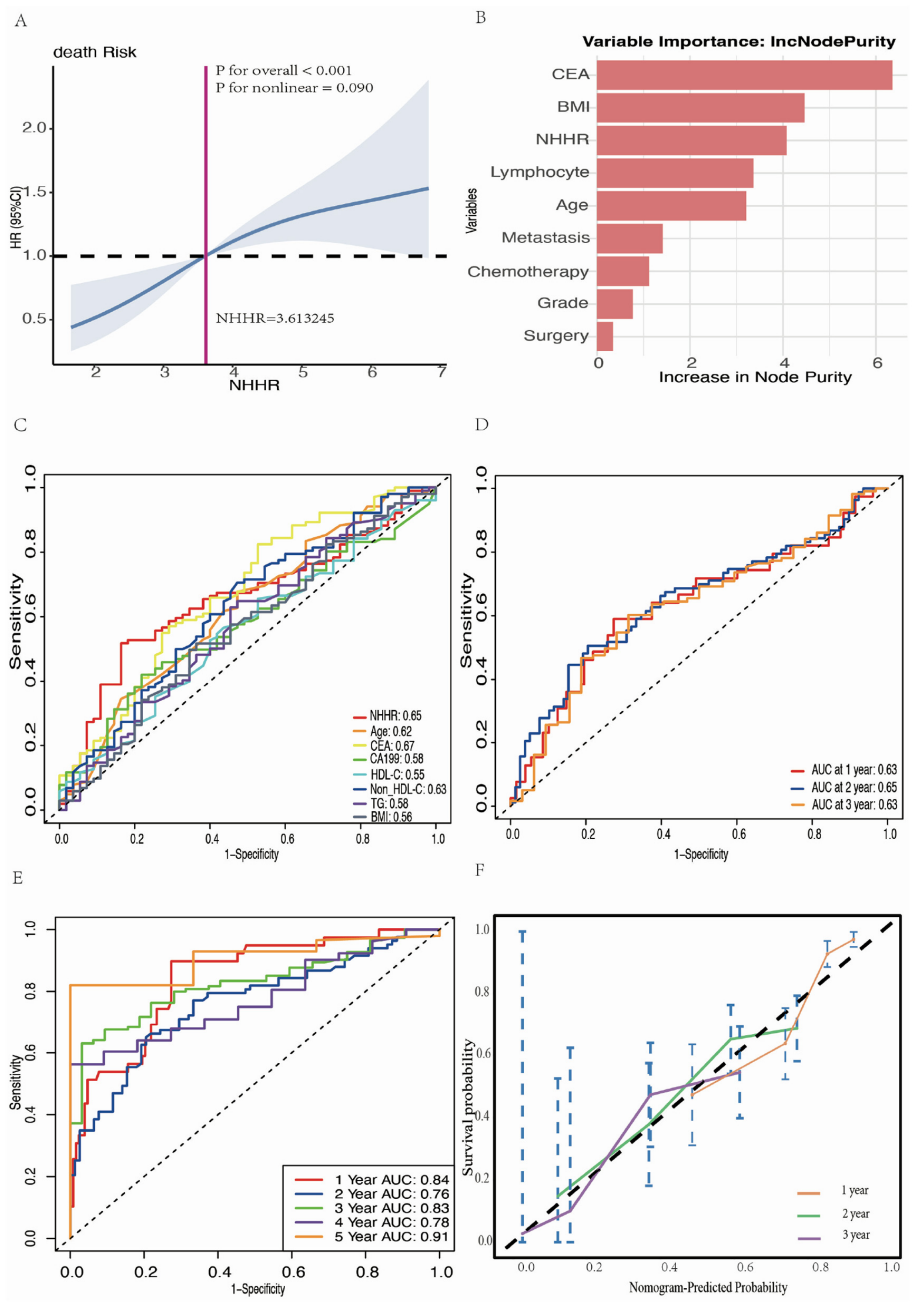
Comprehensive analysis and modeling assessment of the predictive effect of NHHR in mortality risk in RAS-mutant mCRC patients. **(A)** Multivariate RCS analysis of the association between NHHR and risk of death in patients with RAS-mutant mCRC. **(B)** The ranking results of important candidate features obtained through the analysis of Random Forest algorithm. **(C)** ROC curves and AUC values for the prediction of death by different candidate variables. **(D)** Time-dependent ROC curves and time-dependent AUC values for NHHR prediction of death. **(E)** Time-dependent ROC curves were generated to predict mortality using multivariate Cox regression models. Statistical adjustments were made for NHHR, age, BMI, lymphocyte count, CEA levels, tumor grade, metastatic site, chemotherapy, and surgery. **(F)** Calibration curves for the final Cox regression model.

The restricted cubic spline analysis, adjusted for covariates (Figure 7A), further confirmed a significant positive association between NHHR and the hazard of death in patients with RAS-mutant mCRC (overall *P* < 0.001, non-linear *P* = 0.090). In the Gini importance analysis derived from the random forest model, NHHR was identified as one of the most influential variables (Figure 7B). Other variables with high importance included BMI and CEA levels, indicating their substantial contribution to the predictive performance of the model. In the evaluation of predictive accuracy for 2-year survival, the AUC for NHHR was 0.65, second only to CEA (AUC = 0.67), and superior to other covariates (Figure 7C). Moreover, NHHR demonstrated notable temporal stability in predictive performance, with AUC values of 0.63, 0.65, and 0.63 for 1-, 2-, and 3-year survival, respectively (Figure 7D).

### Nomogram construction and model evaluation

Figure 7E presents the time-dependent ROC curves of the final Cox regression model, evaluating its predictive performance at different times. The AUC for these time points were 0.84 at 1 year, 0.76 at 2 years, 0.83 at 3 years, 0.78 at 4 years, and 0.91 at 5 years, indicating that the model possesses strong discriminatory power. Figure 7F displays the calibration curves of the model, showing the agreement between the predicted and actual survival probabilities at 1, 2, and 3 years of follow-up. The results demonstrated a high level of concordance between predicted and observed values.

**Figure 8:**
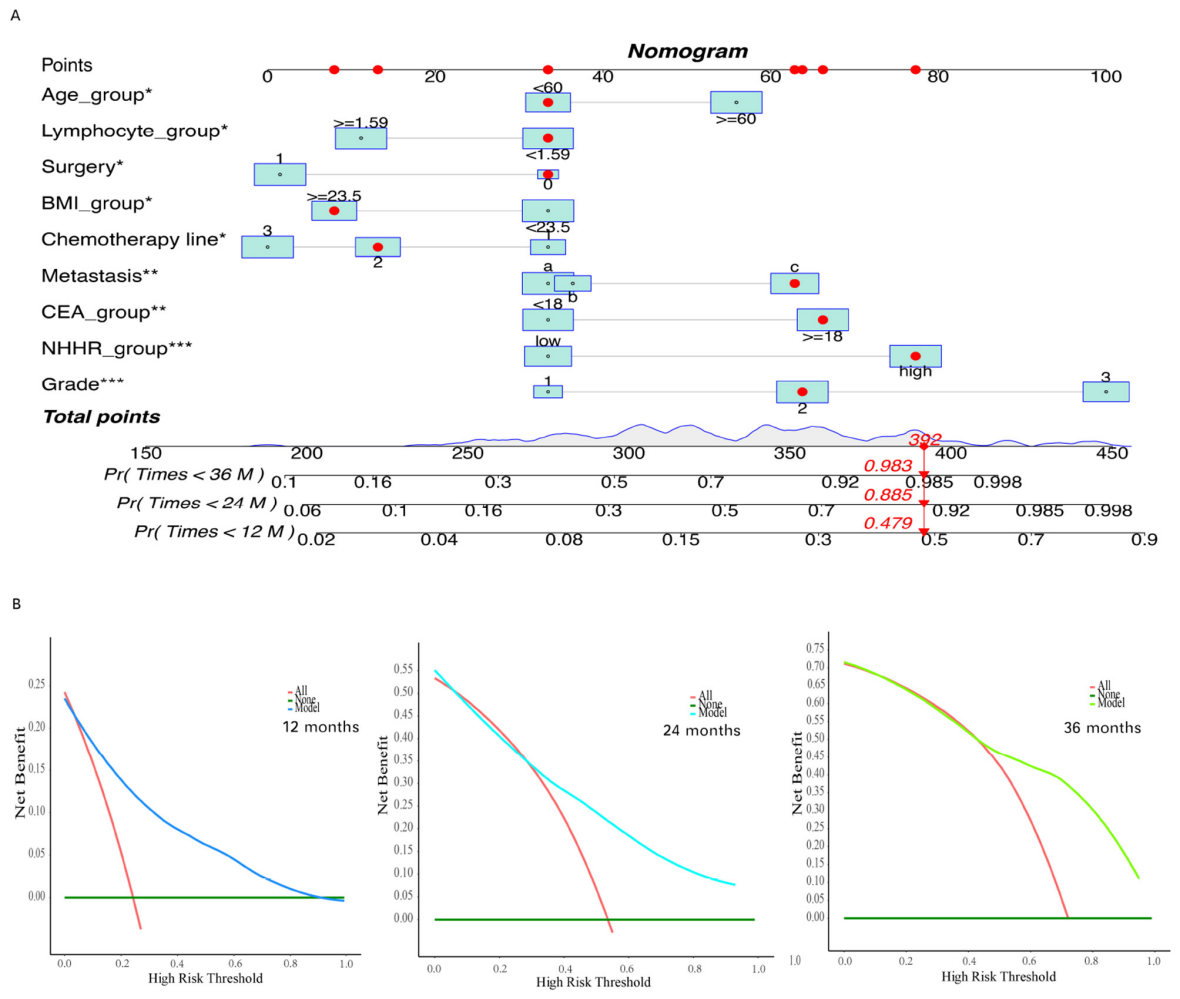
Construct and evaluate a nomogram for survival analysis based on the Cox proportional hazards model. **(A)** The constructed nomogram based on the filtered variables. **(B)** Decision curve for the final Cox regression model. The asterisks (*, **, ***) next to the variable names indicate the levels of statistical significance in the multivariate Cox regression model: *P < 0.05, **P < 0.01, and ***P < 0.001.

To further translate the predictive model into a clinically applicable tool, we constructed a nomogram to visually represent the contribution of each variable to patient outcomes and to support individualized risk prediction. The nomogram illustrates the point assignment for each predictor, with the total score corresponding to the estimated survival probabilities at specific time points (e.g., 12, 24, and 36 months). Figure 8A provides an example of how to estimate the survival probability for an individual patient using the nomogram (indicated by red dots and arrows). The DCA results (Figure 8B) illustrate the net benefit of the model across a range of threshold probabilities. Compared to the strategies of assuming all patients experience the event (red line) or none do (green line), the model consistently yielded greater net benefit across most threshold values. These findings suggest that the model offers good accuracy in predicting patient outcomes at 12, 24, and 36 months, with particularly strong performance at higher threshold probabilities.

In the external validation cohort, patients in the high NHHR group showed significantly worse overall survival compared to those in the low NHHR group (log-rank *P* < 0.001; Supplementary Figure S9A). The nomogram-predicted probabilities demonstrated good agreement with the actual survival outcomes at 1, 2, and 3 years, as shown in the calibration plot (Supplementary Figure S9B). The time-dependent ROC curves showed that the model maintained robust predictive performance over time, with AUCs of 0.78, 0.76, 0.81, 0.80, and 0.79 at 12, 24, 36, 48, and 60 months, respectively (Supplementary Figure S9C). DCA further confirmed the clinical utility of the model, indicating net benefit across a range of risk thresholds for predicting 1-year, 2-year, and 3-year (Supplementary Figures S9D–F) survival.

## Discussion

An NHHR cutoff value of 3.45 was identified as the optimal threshold for predicting poor prognosis in patients with RAS-mutant mCRC. As a comprehensive indicator of lipid metabolism, NHHR was found to be positively correlated with TG, LDL-C, and TC, and negatively correlated with HDL-C, reflecting an imbalance in lipid profiles. In addition, NHHR showed significant positive correlations with inflammatory markers in peripheral blood, including white blood cell count, monocytes, neutrophils, and fibrinogen, as well as with tumor markers such as CA199 and CEA, suggesting its potential role in tumor-associated inflammation and progression.

Given its biological and clinical relevance, an NHHR ≥ 3.45 may serve not only as a prognostic marker but also as a clinically actionable threshold for risk stratification in patients with RAS-mutant mCRC. Patients with elevated NHHR may benefit from closer monitoring and potentially from lipid-lowering or anti-inflammatory strategies. NHHR is a modifiable indicator—prior studies have shown that statins and other lipid-lowering agents can reduce non-HDL-C and increase HDL-C, thereby lowering NHHR. Similarly, healthy dietary patterns (e.g., those rich in unsaturated fats, fiber, and plant-based foods) may improve lipid profiles and systemic inflammation. These findings support the potential of NHHR as a target for nutritional or pharmacologic interventions. In our study, the prognostic value of NHHR was further supported by its inclusion in a predictive nomogram, enabling individualized survival estimates. Nonetheless, prospective studies are needed to determine whether modifying NHHR can lead to improved outcomes in this high-risk population.

Notably, while neither HDL-C nor non-HDL-C alone demonstrated statistically significant associations with overall survival in the multivariable analysis, this does not negate their potential relevance. As shown in Supplementary Figure S4, HDL-C exhibited a clear inverse trend with mortality, though the relationship may be non-linear, suggesting a potential threshold effect or saturation point in its protective role. Non-HDL-C showed a weak positive trend with mortality risk. These findings imply that individual lipid markers may exert heterogeneous and potentially opposing prognostic effects, particularly in the context of advanced colorectal cancer, where dysregulated lipid metabolism, systemic inflammation, and tumor progression interact in complex ways. In contrast, the NHHR integrates both pro-atherogenic and anti-atherogenic lipid components, reflecting a more holistic measure of lipid imbalance and chronic inflammation. This integrated nature likely enhances its prognostic power, leading to the linear and statistically significant association observed with all-cause mortality.

Interestingly, a higher proportion of patients in the low NHHR group reported alcohol consumption. This may be explained by existing evidence showing that light-to-moderate alcohol consumption is associated with increased HDL-C and ApoA1 levels, improved insulin sensitivity, and decreased levels of ApoB, TC, TG, LDL-C, and small dense LDL-C, particularly among moderate drinkers (38, 39). Non-HDL-C levels have also been found to be lower in both light and heavy drinkers compared to non-drinkers, with the difference becoming more pronounced with advancing age, and more evident in females (40, 41). Moreover, large-scale longitudinal studies in Chinese populations have shown that alcohol consumption is associated with a slower rate of HDL-C decline and a more favorable trend in the TC/HDL-C ratio over time (42). These combined lipid-altering effects may contribute to an overall reduction in NHHR and partially explain the distribution observed in our cohort.

Regarding the potential impact of competing cardiovascular mortality risks, we acknowledge that dyslipidemia is associated with cardiovascular death, and that NHHR may also be a strong predictor for cardiovascular outcomes. However, several considerations apply in our case: Our cohort comprised homogeneous, high-risk patients with RAS-mutant metastatic colorectal cancer in China. These patients tend to have shorter survival times and higher cancer-specific mortality rates, reducing the likelihood that non-cancer deaths (e.g., cardiovascular events) would dominate as competing risks. Due to the retrospective nature of this study and limitations in follow-up (relying mainly on telephone interviews), precise cause-of-death data were not available for most patients, which limits our ability to formally model competing risks such as cardiovascular or infection-related deaths.

No studies have yet examined the correlation between NHHR and colorectal cancer prognosis. However, in other tumor types, several studies have demonstrated an important link between disorders of lipid metabolism and disease prognosis. For instance, a retrospective study involving patients with non-small cell lung cancer (NSCLC) revealed that those with higher preoperative HDL-C levels who subsequently experienced a decline in HDL-C after chemotherapy had a longer disease-free survival (DFS) (43). Another study indicated that NSCLC patients undergoing video-assisted thoracoscopic surgery (VATS) in the lower non-HDL-C/HDL-C ratio group exhibited a significantly longer median survival time compared to those in the higher non-HDL-C/HDL-C group (59.00 months vs. 52.35 months) (44). This is similar to the results of this study. In the context of obesity-related cancers, elevated non-HDL-C levels have been associated with increased all-cause and cardiovascular mortality, whereas higher HDL-C levels correlate with reduced all-cause and cancer-specific mortality (45). In patients with chronic lymphocytic leukemia and diffuse large B-cell lymphoma, hypocholesterolemia (both HDL-C and LDL-C) was significantly linked to increased mortality (46, 47). A meta-analysis identified serum TC and HDL-C as protective factors for overall survival in cancer patients (48). Similarly, reduced HDL-C levels have been associated with poorer overall survival in breast cancer (49) and squamous cell carcinoma of the lung (50). Notably, among patients with mCRC undergoing anti-PD1 therapy, higher baseline HDL-C levels and early increases in HDL-C were correlated with improved outcomes (11).

A recent large-scale U.S. pan-cancer study reported a J-shaped association between NHHR and all-cause mortality across diverse cancer types (37), which contrasts with the linear positive association observed in our RAS-mutant mCRC cohort (non-linearity *P* = 0.090). This discrepancy may reflect differences in tumor type, cancer stage, follow-up duration, and competing risks. The pan-cancer study included heterogeneous cancers at various stages and causes of death, whereas our study focused on a high-risk, homogenous population with cancer-specific outcomes and a high mortality rate over a shorter follow-up. Additionally, the limited proportion of patients with very low NHHR (< 1.64) in our cohort may explain the absence of a J-shaped curve's lower limb. Ethnic and lifestyle differences between populations may also contribute to the differing prognostic impact of NHHR. These findings suggest that the prognostic value of NHHR may be context-dependent and warrants further validation in diverse cancer populations.

LDL-C is a key component of cell membrane and steroid hormone synthesis, contributing significantly to the proliferation and survival of cancer cells (51). Research suggests that tumor cells sustain abnormal metabolic states in the tumor microenvironment by increasing cholesterol uptake and synthesis, which promotes their proliferation (51). For example, leukocyte immunoglobulin-like receptor B1 (LILRB1) helps protect multiple myeloma cells from ferroptosis by maintaining cholesterol homeostasis (52). Moreover, elevated levels of LDL and oxidized LDL (ox-LDL), along with overexpression of their receptors (e.g., LDLR, LOX-1, and CD36), are closely associated with cancer cell proliferation, invasion, and angiogenesis (53). LDLR not only regulates cancer cell stemness but may also promote tumor progression by inhibiting immune cell proliferation (54). The exogenous lipid transporter CD36, by activating NF-κB, induces the expression of immunosuppressive genes, thereby facilitating immune evasion in acute myeloid leukemia (AML) (55). Meanwhile, neutrophils that absorb ox-LDL exhibit stronger tumor infiltration potential but reduced cytotoxicity against cancer cells (56). Tumor-secreted FGF21 maintains the hyperactivation of the AKT-mTORC1-SREBP1 signaling axis in activated CD8+ T cells, leading to cholesterol biosynthesis and T cell exhaustion (57). Additionally, high cholesterol levels result in decreased IL-2 and TNF-α expression and increased IFN-γ expression, indicating that elevated cholesterol may induce CD8+ T cell exhaustion, contributing to immune escape in CRC (34). High cholesterol also damages the endoplasmic reticulum structure of CD8+ T cells, increasing the expression of endoplasmic reticulum stress molecules CHOP and GRP78, further leading to CD8+ T cell exhaustion (34). The use of cholesterol-lowering drugs (e.g., statins, PCSK9 inhibitors) may play a role in tumor immunotherapy by promoting the conversion of cold tumors to hot tumors, enhancing CD8+ T cell activity, and reducing PD-L1 expression in cancer cells, thus inhibiting tumor progression (58–62).

HDL represents a heterogeneous family comprising various subpopulations that differ in size, density, and composition, carrying not only cholesterol but also triglycerides, phospholipids, sphingolipids, proteins, enzymes, hormones, and microRNAs (63). HDL inhibits tumor growth by promoting reverse cholesterol transport, thereby reducing intracellular cholesterol accumulation in cancer cells (64). It also exerts antioxidant effects, regulates lipid metabolism, and modulates inflammatory responses within the tumor microenvironment, ultimately suppressing cancer cell invasion and metastasis (65). By regulating cholesterol availability in immune cells, HDL integrates innate and adaptive immune functions, influencing immune synapse formation, apoptosis, and oxidative stress (66). In the gastric cancer immune microenvironment, patients with low HDL levels show reduced tertiary lymphoid structure (TLS) density and delayed maturation compared to those with normal HDL levels (67). Studies have also demonstrated that HDL-C levels are associated with specific immune characteristics in CRC patients, particularly enhanced recruitment and activation of CD3+ and CD8+ T cells (68). Additionally, HDL and its components maintain CD4+ T cell homeostasis and regulate T cells through direct and indirect mechanisms, both of which have antitumor effects (69). HDL and its associated components, such as apolipoproteins and sphingosine-1-phosphate (S1P), may play key roles in antitumor processes by modulating immune responses, inhibiting angiogenesis, and inducing apoptosis (70). Conversely, dysfunctional HDL, which provides less protection against lipid peroxidation, may induce cancer cell proliferation and migration (71). Overall, HDL-C not only plays a critical role in lipid metabolism but also participates in immune regulation through multiple mechanisms, exhibiting both antitumor and protumor potential.

The interplay between lipid metabolism and inflammation has been widely reported. Cholesterol accumulation enhances the inflammatory response in myeloid cells, a process mediated by Toll-like receptor signaling, inflammasome activation, and increased production of monocytes and neutrophils (72). Furthermore, adaptive immune cells undergo cholesterol metabolism reprogramming upon activation, which further enhances the inflammatory response (72). While this response helps combat infection, it may exacerbate conditions in chronic metabolic-inflammatory diseases (72). Studies have shown that low HDL levels are closely associated with increased inflammation, oxidative stress, and impaired immune regulation (73). Additionally, elevated LDL levels correlate with increased levels of proinflammatory cytokines, such as IL-6 and TNF-α, which are thought to support tumor growth and metastasis (74).

Chronic inflammation is considered a key factor in the development and progression of CRC (75). Cytokines, chemokines, and other mediators in the inflammatory environment can promote tumorigenesis and metastasis by activating protumor signaling pathways (76). Lymphocytes typically inhibit tumor growth and dissemination, whereas neutrophils and platelets have the capacity to shift from an antitumor to a pro-tumor and pro-metastatic phenotype (77). Higher neutrophil counts and lower lymphocyte counts suggest a persistent non-specific inflammatory response and a relatively deficient immune state, respectively (78). In cases of exacerbated chronic inflammation, elevated levels of neutrophils, monocytes, and fibrinogen are often observed (79, 80), which were positively correlated with NHHR in this study. This indicates that elevated NHHR may reflect an increased inflammatory state in patients, which not only worsens the tumor course but is also closely associated with poor prognosis. Although we hypothesize that NHHR may influence prognosis through inflammatory pathways based on the biological roles of NHHR and neutrophils, this mechanism (e.g., *cytokines*) was not directly explored in our study. Further investigations integrating transcriptomic, proteomic, or experimental approaches are warranted to elucidate the underlying mechanisms.

Despite providing preliminary evidence for the prognostic value of NHHR in mCRC patients, several limitations exist in this study. First, as a retrospective study, although major known confounders were controlled through multivariate adjustment, selection bias and information bias may affect the generalizability of the results. Second, the small sample size and lack of multicenter data limit the robustness and extrapolation of the findings. Moreover, the absence of fundamental experiments to elucidate the biological mechanisms of NHHR complicates the interpretation of its role in prognosis. NHHR may be influenced by short-term lifestyle factors (e.g., diet, exercise), and the study only used baseline data from the initial treatment phase, failing to account for the dynamic changes of biomarkers. It is worth noting that we treated the effects of continuous variables as linear in the analysis and categorized them into higher and lower groups based on cutoff values. However, these variables may exhibit more complex non-linear relationships, and their clinical significance within normal ranges may vary. Advanced patients typically receive multiple treatment regimens, and the simplification of individual differences in chemotherapy may affect the assessment of treatment efficacy. Additionally, the relationship between NHHR and cancer may be partially explained by competing cardiovascular risks, warranting further investigation into NHHR's role in cancer-specific and cardiovascular-specific mortality. Specific RAS mutation subtypes, including codon-level alterations and isoforms may have distinct prognostic implications in colorectal cancer (81, 82). However, our study is limited by incomplete codon-resolved data for these subtypes. With the widespread implementation of NGS, future large-scale prospective studies are expected to acquire comprehensive genomic profiles, facilitating refined characterization of RAS codon-specific mutations and isoforms and enabling deeper insights into their associations with cholesterol metabolism and clinicopathological features.

## Conclusion

NHHR may serve as a potential prognostic biomarker for metastatic colorectal cancer. Its significant association with survival outcomes highlights the need for further investigation into the mechanisms linking lipid metabolism and cancer progression. This finding also opens new avenues for targeted strategies in personalized treatment. Incorporating lipid profile analysis into routine clinical assessments could provide valuable prognostic insights and aid in tailoring individualized treatment plans for patients with RAS-mutant mCRC.

## Data Availability

The raw data supporting the conclusions of this article will be made available by the authors, without undue reservation.
