# Supplementary material for: Prognostic value of the non-high-density lipoprotein cholesterol to high-density lipoprotein cholesterol ratio (NHHR) in patients with RAS-mutant metastatic colorectal cancer
Source: Front Nutr. 2025 Oct 31;12:1617930. doi: 10.3389/fnut.2025.1617930 (PMC12617302; doi:10.3389/fnut.2025.1617930)
Supplement: Supplementary file 1 [file Data_Sheet_1.pdf]

Supplementary Information to:

Prognostic Value of the Non-High-Density Lipoprotein Cholesterol to High-Density Lipoprotein Cholesterol Ratio (NHHR) in Patients With RAS-Mutant Metastatic Colorectal Cancer

Wenxia Xie[1], Huizhuo Liu[1], Yunjiao Shi[1], Bingxin Zhang[2], Ruoyun Wang[1], Shayan Wang[2], Bin Liang[1]*

Affiliations:

[1]Department of Medical Oncology, First Affiliated Hospital of Wenzhou Medical University, Wenzhou 325000, China;

[2]Department of Internal Medicine, First Affiliated Hospital of Wenzhou Medical University, Wenzhou 325000, China

*__Correspondence Author:__

Prof. Bin Liang, Department of Medical Oncology, the First Affiliated Hospital of Wenzhou Medical University, Wenzhou 325000, China; E-mail: liangbin1992@163.com

Supporting Information

A list of Supporting Information

Supplementary Figure S1. Distribution of RAS mutations in metastatic colorectal cancer (**Page 4**)

Supplementary Table S1. Normality of continuous variables was assessed using the Kolmogorov-Smirnov test (**Page 5**)

Supplementary Figure S2. Correlation of NHHR with other clinical indicators (**Page 6**)

Supplementary Figure S3. A box plot showing the effect of different clinical characteristics on NHHR (**Page 7**)

Supplementary Figure S4. The nonlinear relationship between other lipid metabolism, tumor markers, peripheral blood white blood cells and mortality rate of RAS-mutant mCRC patients (**Page 7**)

Supplementary Figure S5. K-M curves for high and low NHHR groups stratified by Sex, Age, BMI, hypertension, diabetes mellitus, smoking, alcohol consumption, tumor grade, primary tumor site, metastatic site, initial symptoms, surgery, chemotherapy, radiotherapy, CEA, and lymphocyte levels (**Page 8**)

Supplementary Figure S6. Impact of lipid-lowering drug use and RAS mutation subtype on overall survival in patients with metastatic colorectal cancer (**Page 9**)

Supplementary Table S2. Univariate Cox analysis of NHHR and other clinical characteristics with survival in patients with RAS-mutant mCRC (**Page 9-10)**

Supplementary Table S3. Multivariate Cox proportional hazards model results: Model 1 (**Page 10-11**)

Supplementary Table S4. Multivariate Cox proportional hazards model results: Model 2 (**Page 11**)

Supplementary Table S5. Multivariate Cox proportional hazards model results: Model 3 (**Page 12**)

Supplementary Table S6. Variance Inflation Factor for the independent variable (**Page 13**)

Supplementary Figure S7. Variable selection and importance assessment (**Page 13**)

Supplementary Figure S8. The association between NHHR and mortality risk in RAS-mutant mCRC patients: a subgroup analysis (**Page 14**)

Supplementary Figure S9. External validation of the NHHR-based prognostic model in the validation cohort (**Page 15**)

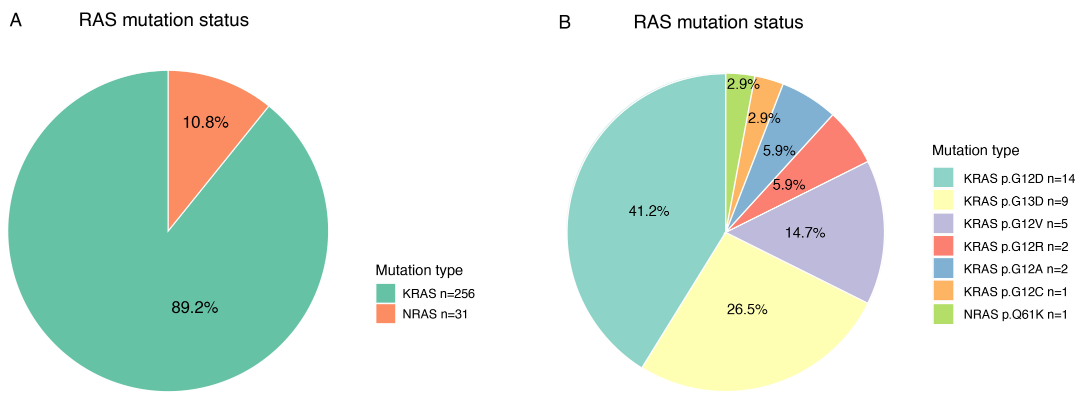

Supplementary Figure S1. Distribution of RAS mutations in metastatic colorectal cancer. (A) Proportion of KRAS and NRAS mutations in all patients. (B) Codon-level mutation spectrum in a subset of patients with available data. Data are presented as counts (n) and percentages.

Supplementary Table S1. Normality of continuous variables was assessed using the Kolmogorov-Smirnov test.

Variable	W	P
NHHR	0.908028092	8.44684E-09
Age	0.986158188	0.09322997
Times	0.95286489	1.9253E-05
Status	0.509569016	1.54986E-21
PNI	0.980314666	0.016814272
BMI	0.991828346	0.452673428
CEA	0.182136462	1.12893E-26
CA199	0.214340155	3.0021E-26
Alkaline phosphatase	0.58118056	4.54802E-20
TC	0.950445082	1.16603E-05
TG	0.841721225	3.00302E-12
LDL	0.936841729	8.60115E-07
HDL	0.976706512	0.006063722
Non-HDL	0.942713163	2.54299E-06
Globulin	0.973469943	0.002514259
Albumin	0.980378247	0.017124918
Total bilirubin	0.833930974	1.38322E-12
BUN	0.950847956	1.26646E-05
Cr	0.920136331	5.21353E-08
AST	0.897593139	1.97484E-09
ALT	0.854891942	1.18398E-11
GBG	0.84928277	6.53601E-12
Uric acid	0.987306075	0.130581878
K	0.982822698	0.034868751
Na	0.973564847	0.002578766
Cl	0.964856007	0.000283841
Hemoglobin	0.977358111	0.007268891
WBC	0.938942957	1.25939E-06
Monocyte	0.916286935	2.87345E-08
Plt	0.894418295	1.29296E-09
Lymphocyte	0.974056305	0.002941625
RDW	0.816271236	2.60644E-13
Neutrophils	0.892604578	1.01876E-09
Fibrinogen	0.952841933	1.91605E-05
D-dimer	0.573900107	3.16447E-20
AFP	0.765082867	3.61841E-15
Creatine kinase	0.8969084	1.80118E-09

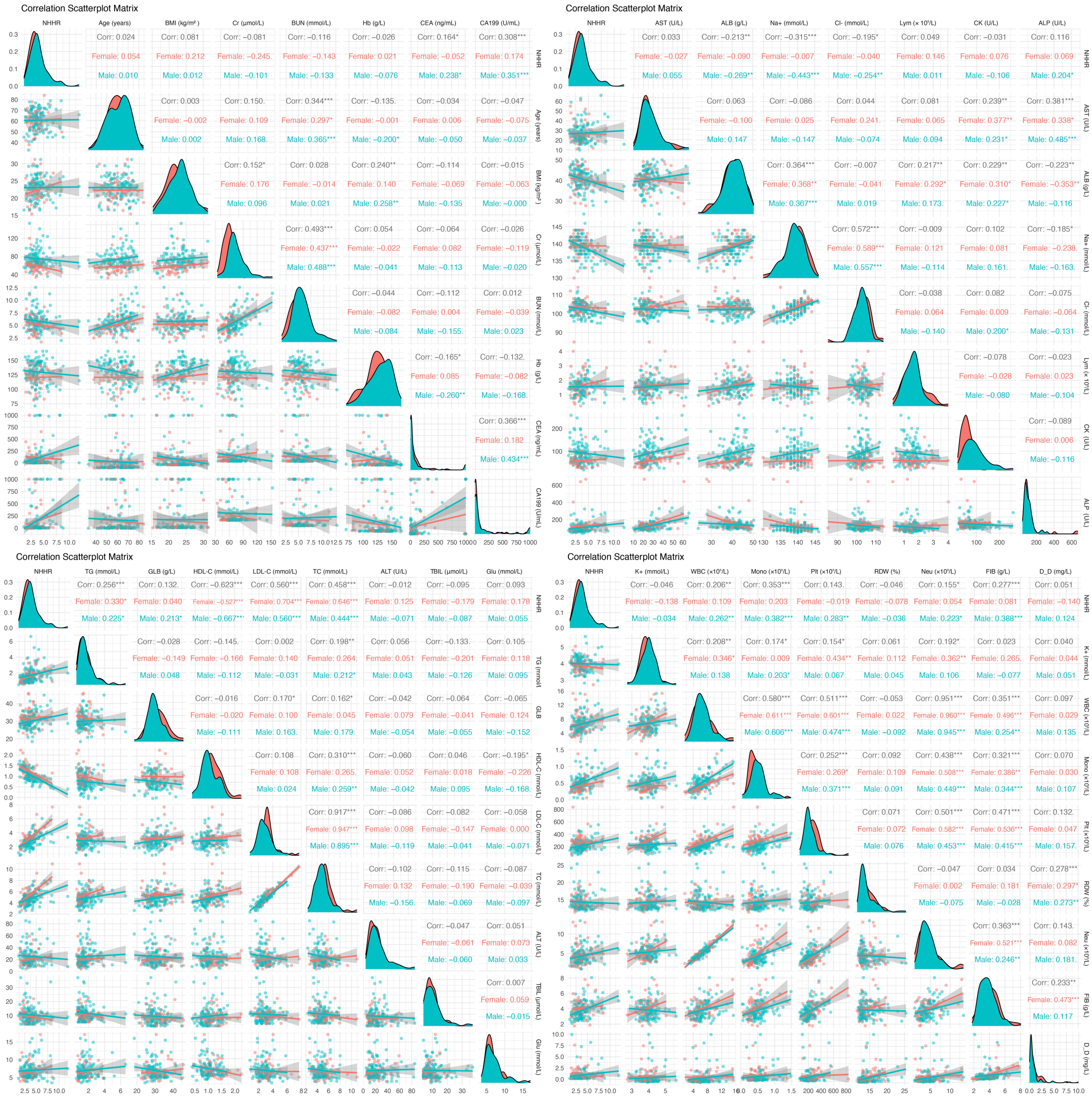

Supplementary Figure S2. Correlation of NHHR with other clinical indicators. The Pearson or Spearman correlation analysis was used to assess the associations between NHHR and other clinical indicators. Data in the graphs are expressed as correlation coefficients, and * labels the significance level. Significance levels were defined as *p < 0.05, **p < 0.01, ***p < 0.001.

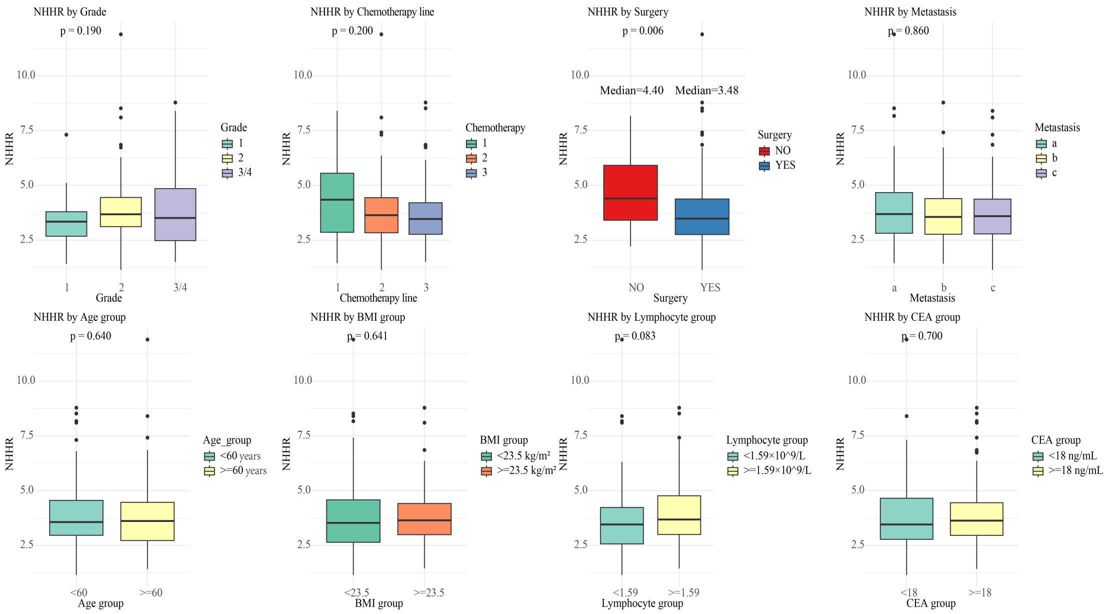

Supplementary Figure S3. A box plot showing the effect of different clinical characteristics on NHHR.

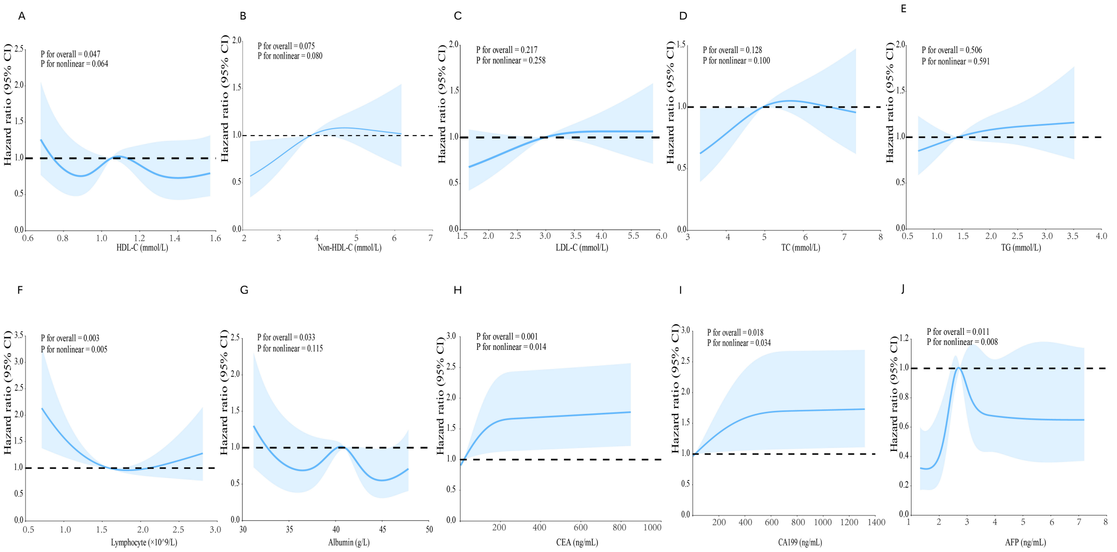

Supplementary Figure S4. The nonlinear relationship between other lipid metabolism, tumor markers, peripheral blood white blood cells and mortality rate of RAS-mutant mCRC patients.

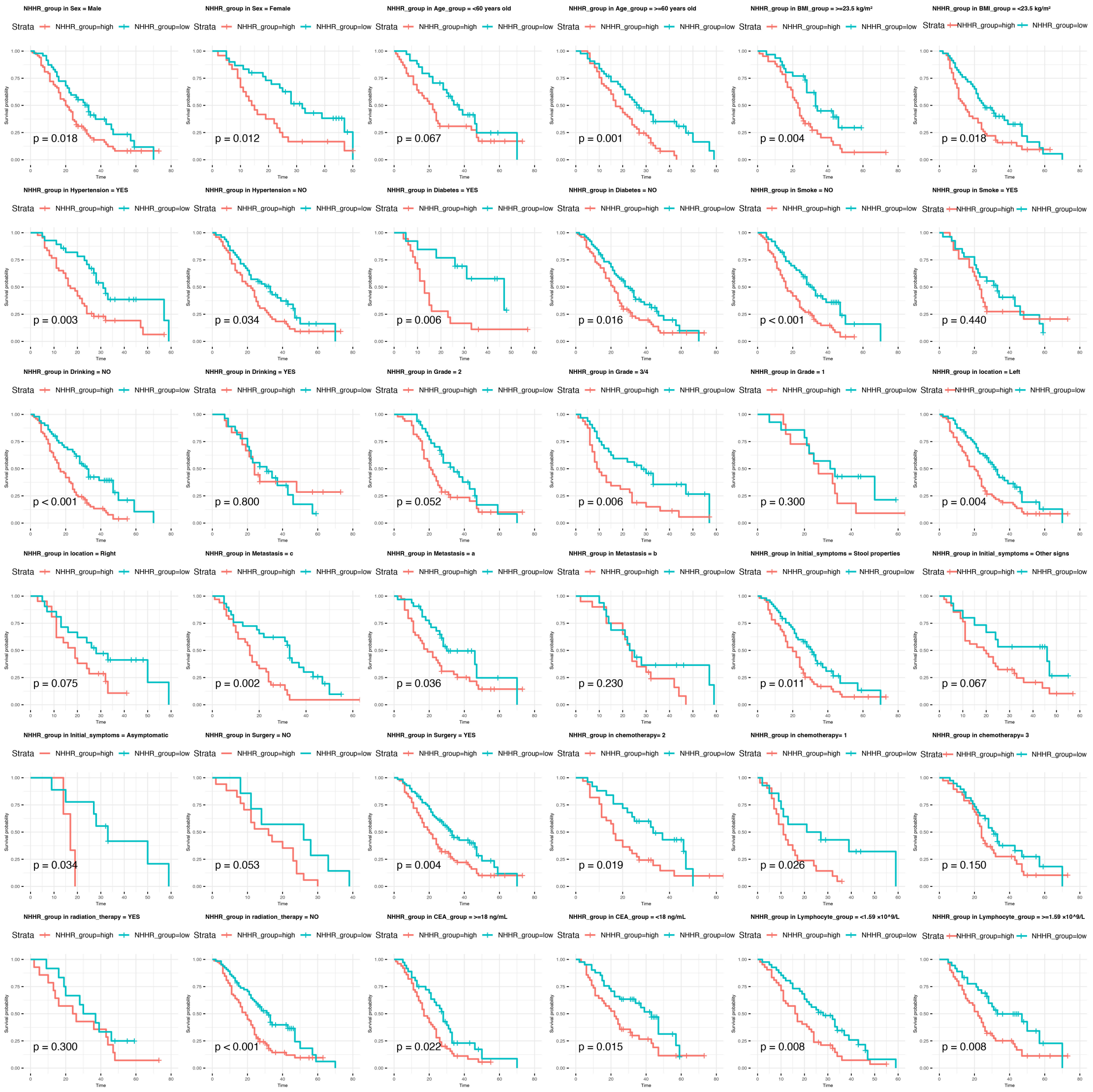

Supplementary Figure S5. K-M curves for high and low NHHR groups stratified by Sex, Age, BMI, hypertension, diabetes mellitus, smoking, alcohol consumption, tumor grade, primary tumor site, metastatic site, initial symptoms, surgery, chemotherapy, radiotherapy, CEA, and lymphocyte levels.

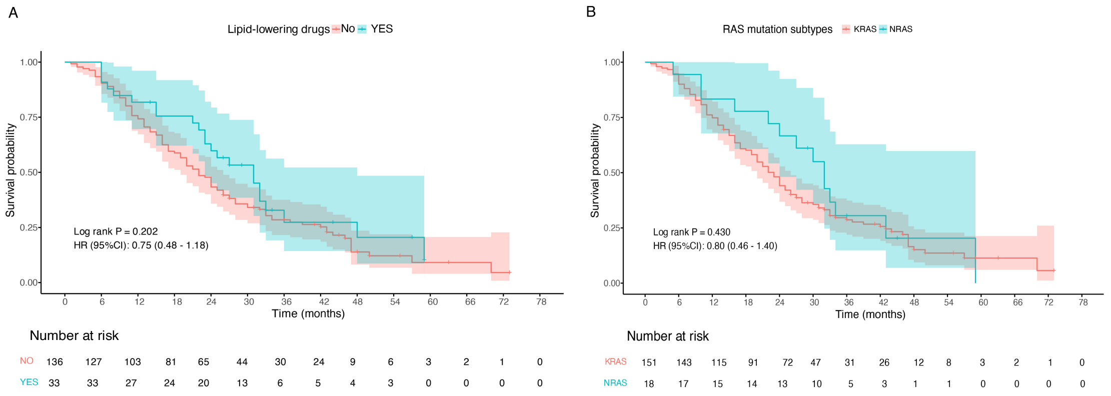

Supplementary Figure S6. Impact of lipid-lowering drug use and RAS mutation subtype on overall survival in patients with metastatic colorectal cancer.

(A) Kaplan–Meier survival curves stratified by lipid-lowering drug use (Yes vs. No).
(B) Kaplan–Meier survival curves stratified by RAS mutation subtype (KRAS vs. NRAS).

Abbreviations: HR, hazard ratio; CI, confidence interval; RAS, rat sarcoma viral oncogene homolog; KRAS, Kirsten rat sarcoma viral oncogene; NRAS, neuroblastoma RAS viral oncogene.

Supplementary Table S2. Univariate Cox analysis of NHHR and other clinical characteristics with survival in patients with RAS-mutant mCRC.

Variables	P value	HR (95%CI)	Variables	P value	HR (95%CI)
NHHR	0.005	1.15 (1.05, 1.27)	Drinking		
Age	0.005	1.03 (1.01, 1.05)	NO		1.00 (Reference)
Sex			YES	0.047	0.66 (0.44, 1.00)
Female		1.00 (Reference)	Radiation therapy		
Male	0.956	1.01 (0.70, 1.47)	NO		1.00 (Reference)
Grade			YES	0.282	0.78 (0.49, 1.23)
1		1.00 (Reference)	BMI	0.05	0.95 (0.90, 1.00)
2	0.521	1.18 (0.71, 1.98)	TC	0.207	1.08 (0.96, 1.21)
3/4	0.073	1.63 (0.96, 2.76)	TG	0.285	1.10 (0.92, 1.31)
location			LDL-C	0.152	1.11 (0.96, 1.29)
Left half of the colon		1.00 (Reference)	HDL-C	0.232	0.69 (0.38, 1.26)
Right half of the colon	0.923	1.02 (0.68, 1.53)	GLB	0.541	1.01 (0.97, 1.05)
Metastasis			ALB	0.057	0.97 (0.93, 1.00)
a		1.00 (Reference)	TBIL	0.561	1.01 (0.98, 1.03)
b	0.362	1.24 (0.78, 1.94)	BUN	0.637	1.02 (0.93, 1.12)
c	0.059	1.46 (0.98, 2.14)	Cr	0.282	1.01 (1.00, 1.02)
Initial symptoms			AST	0.084	1.01 (1.00, 1.03)
Changes in stool properties		1.00 (Reference)	ALT	0.945	1.00 (0.99, 1.01)

Other signs	0.892	0.97 (0.66, 1.43)	Glu	0.374	1.03 (0.96, 1.11)
Asymptomatic	0.697	0.88 (0.46, 1.71)	UA	0.277	1.00 (1.00, 1.00)
Surgery			K^+	0.236	0.79 (0.54, 1.17)
NO		1.00 (Reference)	Na^+	0.007	0.92 (0.86, 0.98)
YES	<0.001	0.44 (0.28, 0.69)	Cl^-	0.002	0.93 (0.89, 0.98)
Chemotherapy			Hb	0.312	1.00 (0.99, 1.00)
First-line		1.00 (Reference)	WBC	0.363	1.03 (0.96, 1.11)
Second-line	0.059	0.64 (0.40, 1.02)	Mono	0.273	1.52 (0.72, 3.19)
More than three lines	0.003	0.51 (0.33, 0.80)	Plt	0.763	1.00 (1.00, 1.00)
Hypertension			Lym	0.084	0.76 (0.55, 1.04)
NO		1.00 (Reference)	RDW	0.962	1.00 (0.93, 1.07)
YES	0.619	1.09 (0.77, 1.55)	Neu	0.164	1.06 (0.98, 1.14)
Diabetes			FIB	0.115	1.13 (0.97, 1.31)
NO		1.00 (Reference)	D-D	0.337	1.05 (0.95, 1.15)
YES	0.914	1.03 (0.65, 1.62)	CEA	0.003	1.00 (1.00, 1.00)
Smoke			CA199	0.05	1.00 (1.00, 1.00)
NO		1.00 (Reference)	AFP	0.161	1.05 (0.98, 1.12)
YES	0.131	0.75 (0.51, 1.09)	CK	0.085	1.00 (0.99, 1.00)
RAS mutation subtypes			ALP	<0.001	1.01 (1.00, 1.01)
KRAS		1.00 (Reference)	Lipid-lowering drugs		
NRAS	0.438	0.80 (0.46 ~ 1.40)	NO		1.00 (Reference)
			YES	0.212	0.75 (0.48 ~ 1.18)

a: Metastasis to a single distant organ or site (e.g., liver, lung), limited to one location;
b: Metastasis to multiple distant organs or sites, without peritoneal involvement; c: Metastasis to one or more distant organs with peritoneal involvement.

Supplementary Table S3. Multivariate Cox proportional hazards model results: Model 1

**Variables**	**P**	**HR (95%CI)**	**Variables**	**P**	**HR (95%CI)**
NHHR	0.002	1.17 (1.06 ~ 1.30)	Smoke		
Age	0.003	1.03 (1.01 ~ 1.05)	NO		1.00 (Reference)
Sex			YES	0.415	0.77 (0.41 ~ 1.44)
Female		1.00 (Reference)	Drinking		
Male	0.392	1.20 (0.79 ~ 1.84)	NO		1.00 (Reference)
Diabetes			YES	0.620	0.85 (0.44 ~ 1.63)
NO		1.00 (Reference)	Lipid lowering drugs		
YES	0.986	1.00 (0.62 ~ 1.60)	NO		1.00 (Reference)
Hypertension			YES	0.057	0.61 (0.37 ~ 1.01)
NO		1.00 (Reference)	BMI (kg/m^2)	0.049	0.94 (0.89 ~ 0.99)
YES	0.472	1.16 (0.77 ~ 1.74)			

Model 1 was adjusted for demographic characteristics and comorbidities, including NHHR, age, sex, BMI, hypertension, diabetes, Lipid-lowering drugs and smoking/alcohol consumption history.

Supplementary Table S4. Multivariate Cox proportional hazards model results: Model 2

**Variables**	**P**	**HR (95%CI)**	**Variables**	**P**	**HR (95%CI)**
NHHR	0.005	1.17 (1.05 ~ 1.31)	Grade		
Age	0.002	1.03 (1.01 ~ 1.05)	Grade 1		1.00 (Reference)
Sex			Grade 2	0.254	1.40 (0.78 ~ 2.50)
Female		1.00 (Reference)	Grade 3/4	0.002	2.64 (1.42 ~ 4.89)
Male	0.159	1.39 (0.88 ~ 2.20)	Treatment Total		
Diabetes			First-line		1.00 (Reference)
NO		1.00 (Reference)	Second-line	0.086	0.63 (0.37 ~ 1.07)
YES	0.133	0.67 (0.40 ~ 1.13)	More than three lines	0.008	0.49 (0.29 ~ 0.83)
Hypertension			Surgery		
NO		1.00 (Reference)	NO		1.00 (Reference)
YES	0.373	1.21 (0.79 ~ 1.86)	YES	0.039	0.59 (0.35 ~ 0.97)
Smoke			RAS mutation subtypes		
NO		1.00 (Reference)	KRAS		1.00 (Reference)
YES	0.259	0.69 (0.37 ~ 1.31)	NRAS	0.128	0.62 (0.33 ~ 1.15)
Drinking			Location		
NO		1.00 (Reference)	Left half of the colon		1.00 (Reference)
YES	0.621	0.84 (0.42 ~ 1.67)	Right half of the colon	0.919	1.03 (0.60 ~ 1.77)
Lipid lowering drugs			Initial symptoms		
NO		1.00 (Reference)	Changes in stool properties		1.00 (Reference)
YES	0.111	0.65 (0.38 ~ 1.10)	Other signs	0.813	0.94 (0.57 ~ 1.55)
BMI (kg/m^2)	0.142	0.96 (0.90 ~ 1.02)	Asymptomatic	0.556	0.79 (0.36 ~ 1.74)
Metastasis			Radiation Therapy		
a		1.00 (Reference)	NO		1.00 (Reference)
b	0.709	0.91 (0.56 ~ 1.49)	YES	0.845	0.95 (0.56 ~ 1.60)
c	0.014	1.69 (1.11 ~ 2.56)			

a: Metastasis to a single distant organ or site (e.g., liver, lung), limited to one location; b: Metastasis to multiple distant organs or sites, without peritoneal involvement; c: Metastasis to one or more distant organs with peritoneal involvement.

Supplementary Table S5. Multivariate Cox proportional hazards model results: Model 3

Variables	P	HR (95%CI)	Variables	P	HR (95%CI)
NHHR	0.003	1.19 (1.06 ~ 1.34)	Grade		
Age	0.002	1.03 (1.01 ~ 1.06)	Grade 1		1.00 (Reference)
Sex			Grade 2	0.335	1.33 (0.74 ~ 2.38)
Female		1.00 (Reference)	Grade 3/4	0.003	2.59 (1.38 ~ 4.85)
Male	0.187	1.36 (0.86 ~ 2.16)	Treatment Total		
Diabetes			First-line		1.00 (Reference)
NO		1.00 (Reference)	Second-line	0.062	0.60 (0.35 ~ 1.03)
YES	0.157	0.69 (0.41 ~ 1.15)	More than three lines	0.008	0.47 (0.27 ~ 0.82)
Hypertension			Surgery		
NO		1.00 (Reference)	NO		1.00 (Reference)
YES	0.551	1.14 (0.74 ~ 1.77)	YES	0.090	0.61 (0.35 ~ 1.08)
Smoke			RAS mutation subtypes		
NO		1.00 (Reference)	KRAS		1.00 (Reference)
YES	0.286	0.70 (0.36 ~ 1.35)	NRAS	0.216	0.66 (0.34 ~ 1.27)
Drinking			Location		
NO		1.00 (Reference)	Left half of the colon		1.00 (Reference)
YES	0.547	0.81 (0.40 ~ 1.63)	Right half of the colon	0.921	1.03 (0.59 ~ 1.79)
Lipid lowering drugs			Initial symptoms		
NO		1.00 (Reference)	Changes in stool properties		1.00 (Reference)
YES	0.107	0.65 (0.38 ~ 1.10)	Other signs	0.804	0.94 (0.56 ~ 1.56)
BMI (kg/m^2)	0.253	0.96 (0.90 ~ 1.03)	Asymptomatic	0.651	0.83 (0.37 ~ 1.86)
Metastasis			Radiation Therapy		
a		1.00 (Reference)	NO		1.00 (Reference)
b	0.762	0.93 (0.57 ~ 1.51)	YES	0.817	0.94 (0.56 ~ 1.59)
c	0.036	1.59 (1.03 ~ 2.45)	Lymphocyte ($\times 10^9$/L)	0.281	0.82 (0.58 ~ 1.17)
CEA（ng/mL)	0.111	1.00 (1.00 ~ 1.00)	Albumin (g/L)	0.543	1.01 (0.97 ~ 1.06)
CA199 (U/mL)	0.976	1.00 (1.00 ~ 1.00)			

a: Metastasis to a single distant organ or site (e.g., liver, lung), limited to one location; b: Metastasis to multiple distant organs or sites, without peritoneal involvement; c: Metastasis to one or more distant organs with peritoneal involvement.

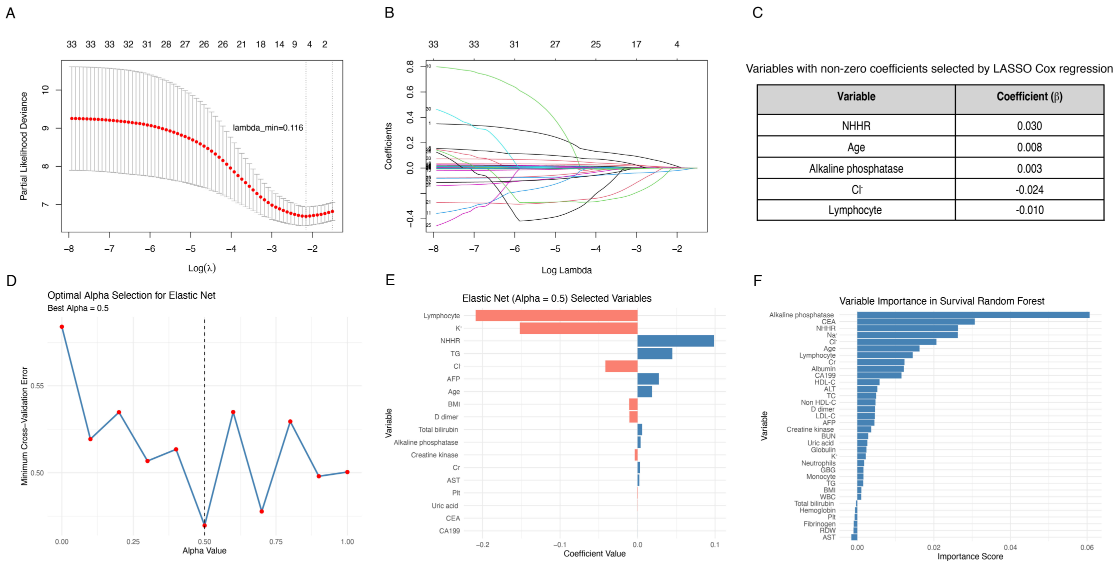

Supplementary Figure S7. Variable selection and importance assessment. (A) Partial likelihood deviance curve for LASSO regression, illustrating optimal λ selection. (B) Coefficient paths of variables across varying λ in LASSO. (C) Final coefficients of selected variables from LASSO. (D) Cross-validation error curve for elastic net alpha selection, with optimal α = 0.5. (E) Coefficients of variables selected by elastic net (α = 0.5). (F) Variable importance from survival random forest analysis.

Supplementary Table S6. Variance Inflation Factor for the independent variable

Variables	VIF	df	standardized VIF
Metastasis	1.066	2	1.016
Age group	1.094	1	1.046
CEA group	1.075	1	1.037
Chemotherapy	1.167	2	1.039
BMI group	1.116	1	1.056
Lymphocyte group	1.055	1	1.027
Grade	1.183	2	1.043
Surgery	1.121	1	1.059
NHHR group	1.068	1	1.033

VIF, Variance Inflation Factor; df, degrees of freedom; NHHR: Non-high-density lipoprotein cholesterol to high-density lipoprotein cholesterol ratio

Variables	n (%)	Lower	Higher	HR (95%CI)	P	P for interaction
		No. of events/ No. of total				
All patients	169 (100.00)	52/77	80/92	2.19 (1.51 ~ 3.18)	<.001	
Age group (years)						0.616
<60	73 (43.20)	21/34	31/39	1.65 (0.87 ~ 3.11)	0.122	
>=60	96 (56.80)	31/43	49/53	2.62 (1.57 ~ 4.37)	<.001	
Metastasis						0.923
a	71 (42.01)	18/32	32/39	2.19 (1.15 ~ 4.20)	0.018	
b	36 (21.30)	12/16	18/20	1.81 (0.70 ~ 4.69)	0.223	
c	62 (36.69)	22/29	30/33	2.70 (1.41 ~ 5.18)	0.003	
Grade						0.302
Grade 1	25 (14.79)	9/14	10/11	1.31 (0.47 ~ 3.64)	0.603	
Grade 2	80 (47.34)	22/31	41/49	2.15 (1.20 ~ 3.84)	0.010	
Grade 3/4	64 (37.87)	21/32	29/32	2.26 (1.19 ~ 4.28)	0.013	
Treatment Total						0.738
First-line	35 (20.71)	10/14	20/21	2.41 (0.95 ~ 6.14)	0.065	
Second-line	58 (34.32)	16/25	28/33	2.16 (1.10 ~ 4.25)	0.026	
More than three lines	76 (44.97)	26/38	32/38	2.14 (1.15 ~ 3.98)	0.017	
Surgery						0.792
NO	24 (14.20)	7/7	17/17	7.52 (1.84 ~ 30.68)	0.005	
YES	145 (85.80)	45/70	63/75	2.13 (1.43 ~ 3.20)	<.001	
BMI group (kg/m²)						0.682
<23.5	96 (56.80)	35/46	44/50	2.20 (1.34 ~ 3.62)	0.002	
>=23.5	73 (43.20)	17/31	36/42	2.08 (1.07 ~ 4.02)	0.030	
CEA group (ng/mL)						0.453
<18	83 (49.11)	23/41	34/42	2.50 (1.41 ~ 4.44)	0.002	
>=18	86 (50.89)	29/36	46/50	2.00 (1.20 ~ 3.35)	0.008	
Lymphocyte group (×10^9 /L)						0.696
<1.59	83 (49.11)	31/41	38/42	2.77 (1.55 ~ 4.94)	<.001	
>=1.59	86 (50.89)	21/36	42/50	2.41 (1.35 ~ 4.28)	0.003	
RAS mutation subtypes						0.762
KRAS	151 (89.35)	47/70	71/81	2.28 (1.53 ~ 3.39)	<.001	
NRAS	18 (10.65)	5/7	9/11	10704.91 (6.47 ~ 17708166.15)	0.014	
Lipid lowering drugs						0.371
NO	136 (80.47)	44/61	65/75	2.02 (1.33 ~ 3.08)	0.001	
YES	33 (19.53)	8/16	15/17	4.55 (1.48 ~ 14.03)	0.008	
Sex						0.037
Female	54 (31.95)	19/30	21/24	4.24 (1.85 ~ 9.71)	<.001	
Male	115 (68.05)	33/47	59/68	1.72 (1.09 ~ 2.73)	0.020	
Location						0.492
Left half of the colon	127 (75.15)	38/56	63/71	2.10 (1.35 ~ 3.28)	0.001	
Right half of the colon	42 (24.85)	14/21	17/21	2.98 (1.15 ~ 7.73)	0.024	

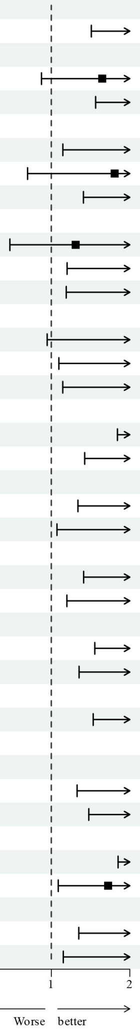

Supplementary Figure S8. The association between NHHR and mortality risk in RAS-mutant mCRC patients: a subgroup analysis. The Cox proportional hazards model was adjusted for NHHR, age, BMI, lymphocytes, CEA, tumor grade, metastatic site, chemotherapy, and surgery.

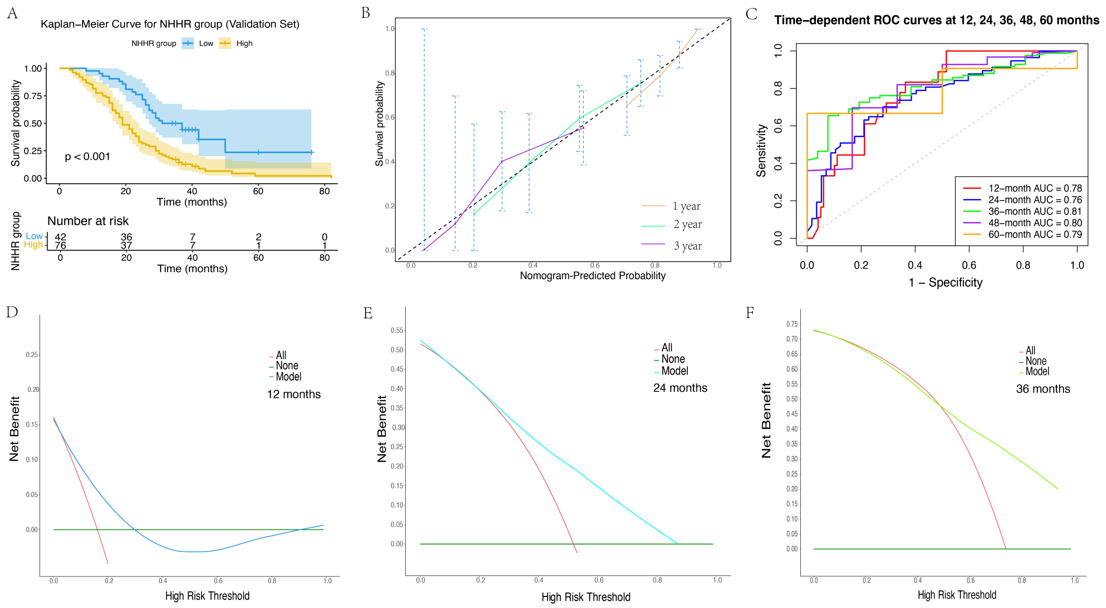

Supplementary Figure S9. External validation of the NHHR-based prognostic model in the validation cohort. (A) Kaplan–Meier survival curves stratified by NHHR level (high vs. low) showed a significantly reduced overall survival in the high NHHR group ($P < 0.001$). (B) Calibration curves at 1-, 2-, and 3-year time points demonstrated good agreement between predicted and observed survival probabilities. (C) Time-dependent ROC curves at 12, 24, 36, 48, and 60 months showed favorable discriminatory performance of the model (AUCs ranging from 0.76 to 0.81). (D–F) Decision curve analysis (DCA) curves at 1 year (D), 2 years (E), and 3 years (F) indicated that the nomogram provided clinical net benefit across a wide range of threshold probabilities compared to the treat-all or treat-none strategies.
